# Supplementary material for: Estimating the effect of moving meat-free products to the meat aisle on sales of meat and meat-free products: A non-randomised controlled intervention study in a large UK supermarket chain
Source: PLoS Med. 2021 Jul 15;18(7):e1003715. doi: 10.1371/journal.pmed.1003715 (PMC8321099; doi:10.1371/journal.pmed.1003715)
Supplement: S1 Appendix — (PDF) [file pmed.1003715.s001.pdf]

Evaluating the effect on sales of moving meat-free products to the meat aisle in a supermarket: protocol for a non-randomised before and after study

Intervention Evaluation Protocol

Versions

Protocol v1.0, 15th April 2019

Protocol v2.0, 4th May 2021 – This version includes the following modifications

1. Add new authors: Carmen Piernas, Peter Scarborough, Richard Stevens
2. Add explanation of Phase II
3. Add detail of sample size calculation and pilot/natural experiment design

Authors

Carmen Piernas
Jennifer Hollowell
Brian Cook
Cristina Stewart
Peter Scarborough
Richard Stevens
Constantinos Koshiaris
Christina Potter
Susan Jebb

Background

Globally, the consumption of meat is rising, driven by population growth and increasing economic development [1]. There is evidence that consuming red and processed meat is associated with adverse health effects, such as an increased risk of developing some forms of cancer [2]. Furthermore, livestock production negatively affects the natural environment through greenhouse gas emissions and other outcomes [3]. Reducing meat consumption can bring health and environmental benefits but there is little research to date on effective behaviour change strategies to achieve this [4].

It is now widely recognized that the physical environments within which food choices are made can exert significant influence on food selection and could potentially to be used to influence consumer demand for meat[5]. Supermarkets are a particularly promising setting for such interventions because of their potential to reach a large number of consumers. However, the evidence on the effectiveness of in-store interventions to reduce meat consumption is lacking [6]. A systematic review of choice architecture interventions to reduce meat consumption found that four

interventions that repositioned meat products to be less prominent at point of purchase showed promising effects on demand for meat, but none of these were delivered in a supermarket setting [6].

There is evidence from other products that in-store positioning can encourage shoppers to switch to healthier foods, and 'cross-category merchandising' (where complementary products are co-located in a store) is an established sales mechanism to increase sales [7, 8]. Research has shown that the provision of meat-free alternatives can help people to reduce their consumption of meat [9], hence positioning meat-free alternative products in store alongside meat products presents a potential opportunity to encourage switching from meat to meat-free alternatives.

This study will evaluate an in-store repositioning intervention in which a major supermarket has moved selected meat-free alternatives from the meat-free section into the meat and poultry aisle in 20 stores.

Research Question

Does repositioning meat-free products to the meat aisle of a supermarket lead to (i) decreases in meat sales and (ii) increases in the sales of meat-free alternatives?

Aims and objectives

The overall aim of this study is to determine whether changing the positioning of meat-free alternatives in supermarket meat/ fish/ poultry to the meat aisles leads to changes in the sales of meat. Additionally it will assess the change in purchases of meat-free alternatives.

The study will focus specifically on the sales of mince, burgers, meatballs and sausages and the corresponding meat-free alternatives.

Secondary aims of this pilot study are (a) to explore the feasibility and practical aspects of delivering and evaluating an in-store intervention in partnership with a commercial retailer, (b) to develop and refine methods of analysis, and (c) to obtain data needed to design and conduct a larger, more definitive in-store intervention study.

Objectives

The study will address the following specific research questions:
At a store level, does the repositioning of meat-free alternatives in the MFP aisle result in:

1. A decrease in sales of the corresponding meat products as measured by volume (units sold) and sales revenue (£)?
2. Changes in sales of meat-free alternatives as measured by volume (units sold) and sales revenue (£)?

The study will also:

3. Develop and pilot methods for monitoring intervention fidelity.
4. Explore and refine analytic methods.
5. Provide initial estimates of potential effect size and other data needed to design a larger, more definitive in-store intervention study.

Study design

A non-randomised controlled before and after study comparing sales/purchasing data in 20 supermarket stores delivering the intervention and a matched sample (provisionally 1:5) of control stores. The intervention will be implemented by stores in the week beginning 27 January 2019. The set-up period (the first week) will be excluded from the analysis of sales.

The study will compare changes in sales of meat and meat-free alternatives in the intervention and control stores across two 12-week time periods: a pre-intervention comparison period (2 September 2018 to 1 December 2018 and the intervention period (3rd February 2019 to 21 April 2019). Subject to data availability, an interrupted time series analysis will also be conducted to evaluate changes in weekly sales of meat and meat-free alternatives associated with the intervention.

The pre-intervention and intervention periods have been selected by the retail partner to avoid months where meat purchases are known to be atypical (e.g. Christmas, 'Veganuary' and summer 'barbeque season').

The impact of the intervention will be evaluated using aggregated weekly store level sales data.

A further evaluation may be conducted using anonymised individual-level purchasing data derived from the retail partner's loyalty card data. This will be covered by a separate protocol.

Selection of intervention stores

The criteria for selecting the intervention stores were set by the retail partner's finance and data teams. To be eligible, intervention stores needed to be supermarkets with excess display bays in the MFP aisle whose inventory volume was not justified by sales or customer volume ('baggy space'). For this pilot study, 20 supermarkets were selected based on a pragmatic balance between the benefits of including more stores in the evaluation with the potential risk of lost sales revenue.

The stores were selected to have a broad spread with regard to the following store characteristics:

- Affluence (based on top 2 Acorn consumer classification categories [10] within 10 minutes' drive, in combination with meat free sales performance (assessed in the 12 week period ending 25 September 2018)
- Store size (Large, Medium, Small, 'Local')

Selection of control stores

The retail partner uses proprietary analytics software to select control stores based on individual –level purchasing data captured by their loyalty card scheme. The software is designed to select control stores based on the following data from up to 104 weeks preceding the intervention on: the store format, and, for the selected product group the seasonal sales trend, sales, visits per customer, spend per visit and customer affluence. The software is designed to identify control stores which have a similar purchasing patter to the intervention stores.

For this study, the software was used to identify 5 matched control stores for each intervention store, based on store customers' patterns of purchase of meat-free alternatives over the 52 weeks preceding the intervention period. A 52 week period rather than 104 weeks was used by the retail partner because the purchasing pattern of meat-free alternatives is undergoing rapid change. The

software does not require all control stores to be unique so stores may be selected as control stores for more than one intervention store.

The research team does not have access to the full details of this matching procedure (since these are confidential), but the suitability of the control sample will be assessed prior to undertaking the main analysis of outcome data (see below). Of particular note, the matching procedure does not explicitly require control stores to have 'baggy' space in the MFP aisle so it will be necessary to verify that the control stores have similar purchasing trends in the pre-intervention period for both meat and meat-free alternatives. If necessary, the selection and matching procedures for the control stores may be modified e.g. removing duplicate control stores from the analysis. Any such changes will be fully documented prior to the main analysis.

Study intervention – PHASE I intervention

Currently, chilled meat products and meat-free alternatives are situated in different aisles of the retail partner's stores. The intervention will involve moving a total of 26 meat-free alternatives (meat-free sausages, meatballs, burgers, steak and mince) from the meat-free section to the MFP aisle. The retail partner's head office has directed store managers in the intervention stores to move these meat-free alternatives to a designated meat-free bay in the MFP aisle and to not stock them anywhere else in stores during the intervention period. Additionally, the meat-free bay in the MFP aisle will have two point of sale (POS) displays in which the following promotional text (developed by the retail partner's staff) will be displayed:

- *Simple swaps - More delicious meat alternatives can be found across the store*
- *Plant power - Easy switches for an alternative source of protein*

In the vast majority (80%) of stores, the meat-free bay in the MFP aisle will replace a bay where meat was previously sold. However, in four of the selected intervention stores, the bay previously contained chilled condiments (three stores) or was unused (one store). Thus in the vast majority of stores, the intervention also involved a reduction in the number of bays devoted to meat sales.

The intervention will run from the week commencing January 27 th to April 21st 2019 (12 weeks excluding the set-up period).

Figure 1 below shows the planogram for meat-free products to be displayed in each of the intervention stores. The meat-free bay will appear at the end (13 stores) or the middle (7 stores) of the MFP aisle in different stores and next to different meat products.

In store checks will be carried out during the intervention period to verify that the intervention has been implemented correctly and to document any protocol deviations or other factors that might have an effect on the evaluation, e.g. stock levels.

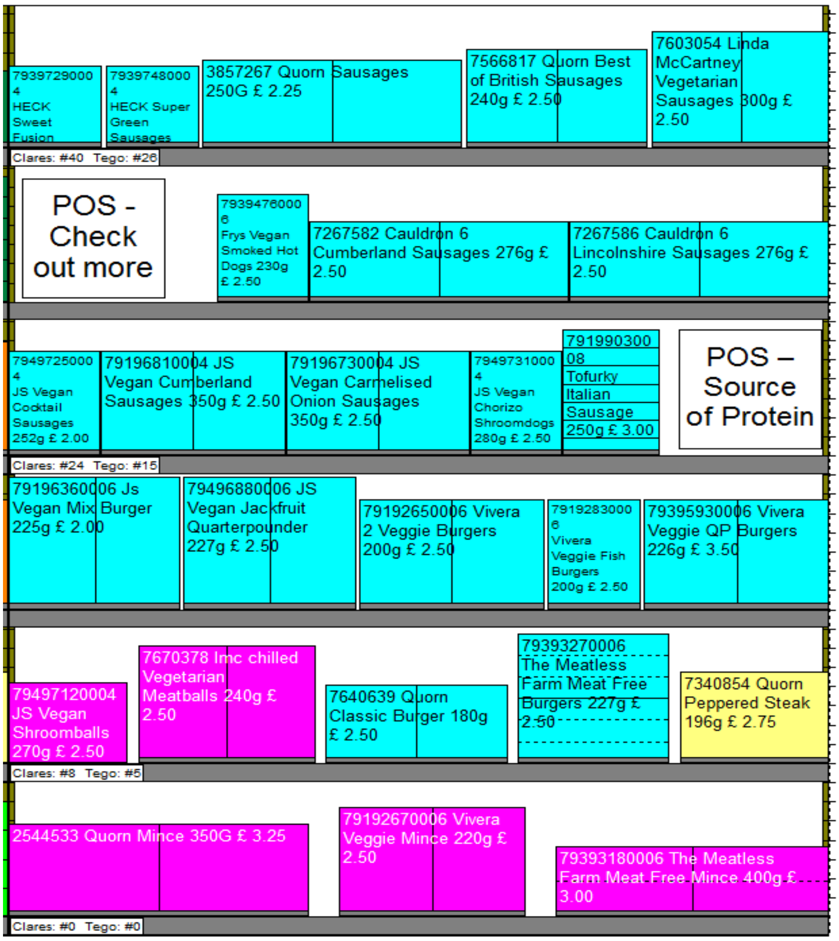

Figure 1: Planogram of meat-free products positioned in the MFP aisle

Study intervention – PHASE II intervention

The intervention was initially planned by the retail partner for 12 weeks until April 2019 (referred to as intervention Phase I). However, 12 of the 20 intervention stores continued the Phase I intervention until December 2019. For the remaining 8 intervention stores, the retailer intensified the intervention (referred to as intervention Phase II) in the week commencing 28th July 2019 by adding a second bay of meat-free products into the meat-free bay within the meat aisle. This second bay of meat-free products included additional meat-free products (e.g. falafels, sausage rolls, tofu, slices) which were stocked alongside the previous 26 products included in the Phase I intervention. These eight stores were selected by the retailer based on each stores having sufficient meat-free products in stock for the second bay. The Phase II intervention continued in these eight stores until December 2019.

Outcome measures

Two measures of sales are available – number of units sold and sales revenue (£) for the product categories of interest. Our primary focus will be on changes in units sold.

Average weekly sales (units and £) will be calculated as the average of weekly sales for the relevant study period i.e. either the comparator or intervention period

In order to be able check that any changes in purchasing are attributable to the intervention and not to confounding, we will also evaluate sales of two products (fresh fish and non-dairy milks) which should be unaffected by the intervention in both intervention and control stores.

All outcome measures are based on sales of the 26 stock keeping units (SKUs) that have been moved into the MFP aisle and on the meat product equivalents.

Primary outcomes

- Average total weekly sales (Units) of mince, burgers, meatballs and sausages (meat)

Secondary outcomes

- Average weekly sales (Units and £) per store of mince (meat)
- Average weekly sales (Units and £) per store of burgers (meat)
- Average weekly sales (Units and £) per store of meatballs (meat)
- Average weekly sales (Units and £) per store of sausages (meat)
- Average total weekly sales (£) of mince, burgers, meatballs and sausages (meat)
- Average weekly sales (Units and £) per store of meat-free alternative mince
- Average weekly sales (Units and £) per store of meat-free alternative burgers
- Average weekly sales (Units and £) per store of meat-free alternative meatballs
- Average weekly sales (Units and £) per store of meat-free alternative sausages
- Average total weekly sales (Units and £) of meat free alternative mince, burgers, meatballs and sausages

Control outcomes

- Average weekly sales (Units and £) per store of fish
- Average weekly sales (Units and £) per store of non-dairy milk

Study procedures

Monitoring

All intervention stores will be visited at least twice during the intervention period to check the fidelity of intervention implementation. Specifically, to check the extent to which:

- The 26 meat-free alternative SKUs have been moved to the meat aisle.
- These 26 SKUs are not on sale elsewhere in the store.
- These 26 SKUs are in stock at the time of the visit.
- The meat-free alternative SKUs designated to be stocked on the middle eye-level shelves are correctly placed.

A sample of control stores will be visited at least once during the intervention period to check the extent to which:

- The meat-free alternatives identified in the protocol are not positioned in the meat aisle but are stocked elsewhere in the store.

The monitors will take photos of the relevant bays to document products on sale, stock levels, presence and position of point of purchase (POS) displays and position of bays within the MFP aisle.

The monitors will record the location of the meat-free alternative bay in the MFP aisle and document any meat-free alternatives found to be out of stock at the time of the visit using a data collection form designed for this purpose.

The monitors will also check for other in store promotions running that might affect sales of meat or meat-free alternatives.

Monitoring frequency may be increased if findings suggest protocol deviations or other issues. A fidelity score will be estimated for each store by investigators blind to the outcome measures.

Statistical methods and data analysis plan

Sample size calculations

Original: This is a pilot study. No statistical power calculations have been conducted and we consider the findings to be of a exploratory nature. Data from this pilot study will be used to inform the design of future intervention evaluation studies.

Revised: The study was initially conceived as a pilot study with the view of estimating the effect sizes for a future trial. However the retailer did not continue their plans to conduct a larger trial. Therefore following the conduct and evaluation of a natural experiment, a power analysis was not conducted and the final number of trial stores with matched control stores was chosen by the retailer.

Data analysis plan

The aim of the analysis is to determine whether there was a change in sales of specific products in the intervention stores, relative to sales in the control stores. However, at the time of designing the study, we do not know whether time trends in sales of meat and meat-free alternatives are similar in the intervention and control stores (which would invalidate analysis methods that assume common trends) or whether the shoppers in the intervention and control stores differ in their propensity to reduce their meat purchases or switch to meat-free alternatives (which could lead to confounding if not controlled for in the analysis).

Preliminary analysis of pre-intervention data

The following preliminary analyses will be conducted using the data listed in Annex A, blinded to the outcome data:

Time trends: We will plot time trends in the study outcomes, by store and overall (intervention vs control) for up to 25 months preceding the start of the intervention. Subject to data availability we will plot weekly sales data. If weekly data are not available we will examine trends by comparing sales data for the time periods January-March 2018 and September-November 2018 and, if possible for the same periods in 2017 i.e. periods corresponding to the study intervention and pre-intervention comparator periods.

We will visually inspect these plots to assess whether time trends are broadly similar in the intervention and control stores and whether there is any evidence of systematic differences by store characteristics, using the store classification data provided by the retail partner. These data include affluence (more affluent, average, less affluent), customer age (younger, older), ethnic mix (White, Asian, Other) and population density (more urban, urban, less urban). Outliers may be investigated.

Propensity to reduce meat purchases and/or switch to meat-free alternatives We will compute a range of store-level measures designed to capture propensity to reduce meat purchase and increase purchases of meat-free alternatives. These will include: change in sales of meat and meat-free alternatives in 'Veganuary' relative to sales in other periods. We will visually explore whether these outcomes appear to differ between intervention and control stores and whether these measures appear to differ by store characteristics.

The data required to conduct these preliminary analyses are summarised in annex A.

Based on these preliminary analyses we will specify and document the variables that are potential confounders.

If time trends appear to differ between the intervention and control stores, we may define a subgroup of stores which we consider to have comparable sales time trends, prior to commencing analysis of the main outcomes.

Analysis strategy

The purpose of the analysis is to describe changes in sales volume of meat products and meat-free alternatives and to test for differences is sales volume between the intervention and control stores, adjusted for differences in sales volume during the pre-intervention comparator period. The analysis will also adjust for store characteristics found to be potential confounders in the preliminary analysis.

Prior to analysing the primary and secondary outcome data, we will run the analysis for control outcome measures (e.g. fish and non-dairy milk sales) to check that sales of these two products do not differ between intervention and control stores, after adjusting for pre-intervention sales and store characteristics.

The main analysis will be conducted using all intervention and control stores. If the two groups are found not to be comparable with regard to time trends, we may additionally perform a subgroup analysis based on stores considered to be comparable. Other exploratory analyses may be conducted but will be described as *post hoc* in the study reports.

Statistical methods

For all outcomes, we will report the number and percentage of observed values, mean and standard deviation, median and inter-quartile range, and range, by arm (intervention vs control) and for the full sample, and by time period (intervention period vs pre-intervention comparator period). The outcome measure (mean weekly units sales) is a count outcome thus analysis will be performed using regression models that deal with count outcomes.

We will use conditional regression to account for the matching of stores. Univariate associations between baseline characteristics (pre-intervention period sales) and outcome (intervention period sales) will be examined with the use of conditional negative binomial regression. Multivariate analysis will be conducted using conditional negative binomial regression adjusted for the covariates (store characteristics) and also the baseline count (pre-intervention period sales)[11].

Results will be presented as incident-density ratios with 95% confidence intervals and p-values. For this pilot study, all statistical tests will be conducted at a 5% significance level, but interpretation of findings for secondary outcomes will be based on consistency of findings in recognition of the risk of false positives associated with multiple testing.

In the event that some stores are selected as control stores for multiple intervention stores, we will run the analysis on the full dataset and conduct a sensitivity analysis to assess whether this affects the findings. If appropriate, duplicates will be removed and the analysis re-run using only unique control stores.

In addition use of interrupted time series will be considered depending on the nature and availability of data in order to examine the effectiveness of the intervention [12].

References

1. York, R. and M.H. Gossard, *Cross-national meat and fish consumption: exploring the effects of modernization and ecological context.* Ecological Economics, 2004. **48**(3): p. 293-302.
2. Afshin, A., et al., *Health effects of dietary risks in 195 countries, 1990–2017: a systematic analysis for the Global Burden of Disease Study 2017.* The Lancet.
3. Steinfeld, H., et al., *Livestock's long shadow: environmental issues and options*. 2006: Food & Agriculture Org.
4. Bianchi, F., et al., *Interventions targeting conscious determinants of human behaviour to reduce the demand for meat: a systematic review with qualitative comparative analysis.* International Journal of Behavioral Nutrition and Physical Activity, 2018. **15**(1): p. 102.
5. Hollands, G.J., et al., *The TIPPME intervention typology for changing environments to change behaviour.* Nature Human Behaviour, 2017. **1**(8): p. 0140.
6. Bianchi, F., et al., *Restructuring physical micro-environments to reduce the demand for meat: a systematic review and qualitative comparative analysis.* The Lancet Planetary Health, 2018. **2**(9): p. e384-e397.
7. Cameron, A.J., et al., *A Systematic Review of the Effectiveness of Supermarket-Based Interventions Involving Product, Promotion, or Place on the Healthiness of Consumer Purchases.* Current Nutrition Reports, 2016. **5**(3): p. 129-138.
8. Thorndike, A.N., et al., *Choice architecture to promote fruit and vegetable purchases by families participating in the Special Supplemental Program for Women, Infants, and Children (WIC): randomized corner store pilot study.* Public health nutrition, 2017. **20**(7): p. 1297-1305.
9. Holloway, T., A.M. Salter, and F.S. McCullough, *Dietary intervention to reduce meat intake by 50% in University students – a pilot study.* Proceedings of the Nutrition Society, 2012. **71**(OCE2): p. E164.
10. CACI, *Acorn User Guide*. 2014.
11. Zheng, H., et al., *A comparison of different ways of including baseline counts in negative binomial models for data from falls prevention trials.* Biometrical journal, 2018. **60**(1): p. 66-78.
12. Bernal, J.L., S. Cummins, and A. Gasparrini, *Interrupted time series regression for the evaluation of public health interventions: a tutorial.* International Journal of Epidemiology, 2016. **46**(1): p. 348-355.

Annex A: Specification of pre-intervention exploratory dataset, main analysis dataset, and list of store characteristic variables

Sales data

1. **Exploratory pre-intervention dataset:** Weekly store-level sales data for the time period January 2017 - January 2019 (inclusive).
2. **Main analysis dataset:** as above but including the intervention period (February – April 2019)

Each row of the dataset to contain data for one week for a single store (or other format to be agreed)

Variables:
Store ID (unique store number)
Store name
Store group (store ID of intervention/test store)
Store category (intervention/control)
Year (2017/2018/2019)
Week (1-52)
Sales (number of units) of mince
Sales (number of units) of burgers
Sales (number of units) of meatballs
Sales (number of units) of sausages
Sales (number of units) of meat-free alternative mince
Sales (number of units) of meat-free alternative burgers[1]
Sales (number of units) of meat-free alternative meatballs
Sales (number of units) of meat-free alternative sausages
Sales (number of units) of fish
Sales (number of units) of non-dairy milk
Sales (£) of mince
Sales (£) of burgers
Sales (£) of meatballs
Sales (£) of sausages
Sales (£) of meat-free alternative mince
Sales (£) of meat-free alternative burgers
Sales (£) of meat-free alternative meatballs
Sales (£) of meat-free alternative sausages
Sales (£) of fish
Sales (£) of non-dairy milk

[1] This category will include 'veggie fish burgers' and meat-free steak alternatives

Store characteristics

For each store:

- Store ID (Unique store number)
- Region
- Location type (retail park, suburban, town centre, etc)
- Affluence (more, less, average)
- Customer age (younger, older)
- Ethnicity of customer base (White, Asian, Other)
- Population Density (More Urban, Urban, Less urban, Rural)
- Store size (Large, Medium, Small, SSLL)
- Whether store had excess space in the MFP aisle in the pre-intervention period (‘Baggy space’)
