# Supplementary material for: Estimating the effect of moving meat-free products to the meat aisle on sales of meat and meat-free products: A non-randomised controlled intervention study in a large UK supermarket chain
Source: PLoS Med. 2021 Jul 15;18(7):e1003715. doi: 10.1371/journal.pmed.1003715 (PMC8321099; doi:10.1371/journal.pmed.1003715)
Supplement: S2 Appendix — (PDF) [file pmed.1003715.s002.pdf]

Evaluating the effect on sales of moving meat-free products to the meat aisle in a supermarket: a non-randomised controlled before and after study

Statistical Analysis Plan

Versions:

Versions 0.1 to 0.5, 14th November 2019 - 10th January 2020

1. Statistical analyses agreed

Version 0.6, 4th May 2021 – This version includes the following modifications

1. Add new authors: Carmen Piernas, Peter Scarborough, Richard Stevens
2. Add explanation of Phase II
3. Add detail of sample size calculation and pilot/natural experiment design

Authors

Carmen Piernas
Jennifer Hollowell
Brian Cook
Cristina Stewart
Peter Scarborough
Richard Stevens
Constantinos Koshiaris
Christina Potter
Susan Jebb

Research Questions and Aims

Does repositioning meat-free products to the meat aisle of a supermarket lead to (i) decreases in meat sales and (ii) increases in the sales of meat-free alternatives?

The overall aim of this study is to determine whether changing the positioning of meat-free alternatives in the meat aisles leads to changes in the sales of meat and meat-free alternatives, including mince, burgers, meatballs and sausages and the corresponding meat-free alternatives.

At a store level, does the repositioning of meat-free alternatives in the meat aisle result in:

1. A decrease in sales of the corresponding meat products as measured by volume (units sold) and sales revenue (£)?
2. Changes in sales of meat-free alternatives as measured by volume (units sold) and sales revenue (£)?

Study design

A non-randomised controlled before and after study comparing sales/purchasing data in active trial stores delivering the intervention and a matched sample (1:5) of control stores.

The intervention has been carried out in 2 phases:

- Phase I – Positioning of meat-free products in meat aisle

 The intervention consists of repositioning meat-free products in the meat aisle from the week commencing January 27th to April 21st 2019 (12 weeks). A total of 20 trial stores implemented this intervention over the specified period, while 88 matching control stores carried out business as usual.

 Although the supermarket pre-specified that the intervention was going to finish in the w/c April 28th 2019, the trial stores maintained the meat-free aisle until the end of the study period (w/c December 1st 2019). For the main analyses, we will consider the intervention period to run for 12 weeks from w/c February 3rd to w/c April 21st 2019.

- Phase II – Addition of extra bay for meat-free products in meat aisle

 The phase II intervention consists of the addition of another bay for meat-free products in the meat aisle from the week commencing July 28th 2019 with no pre-defined end date for the trial. A total of 8 stores from the above trial 20 stores were selected to incorporate the extra unit. The remaining 12 trial stores continued with the phase I intervention, while the 88 matching control stores continued carrying out business as usual. The 8 stores were selected by the retailer based on the number of meat-free products in stock. Of the 20 original intervention stores, 8 had sufficient meat-free products to fill a second bay. Other stores did not have enough for a complete bay and mixing meat and meat-free products in the same bay was seen as unacceptable.

Matching was carried out by the commercial partner and full details of the matching algorithm are not available to the research team. Although matching of 5 control stores to 1 intervention store was sought, in practice some matching groups have fewer than 5 controls. Some stores will be allocated to a single matching group by selecting the matching group with fewest control stores before the data analysis.

Store characteristics

We have the following store characteristics which related to the surrounding population which may be included as covariates in the analysis:

- Affluence: coded as More; Average; Less
- Age group: coded as binary (Older vs Younger)
- Ethnicity: coded as White; Asian; Other
- Area: coded as More Urban; Urban; Less Urban

Outcome measures

Primary outcome

Two measures of sales are available – number of units sold and sales revenue (£) for the product categories of interest. Our primary outcome will be on units sold:

- Average total weekly sales (Units) of mince, burgers, meatballs and sausages (meat)

Average weekly sales (units and £) will be calculated as the average of weekly sales for the relevant study period i.e. either the comparator or intervention period

All outcome measures are based on sales of the 26 stock keeping units (SKUs) that have been moved into the MFP aisle and on the meat product equivalents.

Secondary outcomes

Of major importance is re-assessment of the primary outcome using an alternative measure (£) as well as changes in sales of meat-free alternatives:

- Average total weekly sales (£) of mince, burgers, meatballs and sausages (meat)
- Average total weekly sales (Units) of meat free alternative mince, burgers, meatballs and sausages
- Average total weekly sales (£) of meat free alternative mince, burgers, meatballs and sausages

Additionally, when an effect is identified on one of the above four outcomes, we will further stratify by type of product:

- Average weekly sales (Units and £) per store of mince (meat)
- Average weekly sales (Units and £) per store of burgers (meat)
- Average weekly sales (Units and £) per store of meatballs (meat)
- Average weekly sales (Units and £) per store of sausages (meat)
- Average weekly sales (Units and £) per store of meat-free alternative mince
- Average weekly sales (Units and £) per store of meat-free alternative burgers
- Average weekly sales (Units and £) per store of meat-free alternative meatballs
- Average weekly sales (Units and £) per store of meat-free alternative sausages
- Average weekly sales (Units and £) per store of other meat-free items (e.g. tofu, falafel)

Control outcomes

- Average weekly sales (Units and £) per store of fish
- Average weekly sales (Units and £) per store of non-dairy milk
- Average weekly sales (Units and £) per store of vegetables
- Average weekly sales (Units and £) per store of fruit
- Average weekly sales (Units and £) per store of personal-care products

Statistical methods

Sample size considerations

Original: This is a pilot study. No statistical power calculations have been conducted and we consider the findings to be of a exploratory nature. Data from this pilot study will be used to inform the design of future intervention evaluation studies.

Revised: The study was initially conceived as a pilot study with the view of estimating the effect sizes for a future trial. However the retailer did not continue their plans to conduct a larger trial. Therefore following the conduct and evaluation of a natural experiment, a power analysis was not conducted and the final number of trial stores with matched control stores was chosen by the retailer.

Descriptive analysis of pre-intervention data

We will tabulate store characteristics by intervention vs control group, and test for differences with *t*-test (continuous variables) or chi-squared test (categorical variables);

- Affluence;
- Age group;
- Ethnicity;
- Area;
- "Veganuary" effect (e.g. higher sales of meat-free products in January 2018 and 2019, see below);
- Average sales of <outcome> per week in 12 weeks before baseline.

Normality assumptions for continuous variables will be checked graphically (quantile plots) to determine whether alternative methods (log transformation or if necessary non-parametric methods) are required.

We will compute store-level measures designed to capture propensity to reduce meat purchase and increase purchases of meat-free alternatives: in particular, change in sales of meat and meat-free alternatives in 'Veganuary' relative to sales in other periods.

The data required to conduct these preliminary analyses are summarised in annex A.

Analysis strategy

The purpose of the analysis is to describe changes in sales volume of meat products and meat-free alternatives and to test for differences in sales volume between the intervention and control stores, adjusted for differences in sales volume during the pre-intervention comparator period. The analysis will also adjust for store characteristics found to be potential confounders in the preliminary analysis but we will also explore doing subgroup analyses by store characteristics.

Prior to analysing the primary and secondary outcome data, we will run the analysis for the control outcome measures to check that sales of these two products do not differ between intervention and control stores, after adjusting for pre-intervention sales and store characteristics.

The main analysis will be conducted using all intervention and control stores. Outliers will be investigated and excluded if necessary in a sensitivity analysis. If the intervention and control stores are found not to be comparable with regard to time trends, we may additionally perform a subgroup analysis based on stores considered to be comparable.

All statistical tests will be conducted at a 5% significance level, but interpretation of findings for secondary outcomes will be based on consistency of findings in recognition of the risk of false positives associated with multiple testing.

Statistical methods for primary outcome
Analyses will exclude the first week (to allow time for the intervention to be implemented). The primary analysis will compare weekly units sold in intervention to control stores during the phase I intervention period (w/c 3rd February 2019 to w/c 21 April 2019) using a hierarchical negative binomial model, with fixed effect adjustment for store affluence, store age group, store ethnicity, store area and average units sold per week in 12-week pre-intervention period (w/c 2 September 2018 to w/c 18 November 2018) and a random effects term for matching group.

Statistical methods for other outcomes
Analyses of other outcomes will proceed as above except that the model will be hierarchical Normal for sales (£) outcomes.

Secondary analyses
For both phase I and II trials, the impact of the interventions will be also evaluated using aggregated store level sales data and interrupted time series analysis will be conducted to evaluate changes in weekly sales associated with the interventions using data from w/c 1st August 2017 to w/c 1st December 2019. We will compute a graphical representation of the outcomes with p-values. For evaluation of the Phase I intervention, we will plot (a) the average sales of meat and meat-free products across all 20 intervention stores and (b) the average sales of meat and meat-free products across all 88 control stores, together with fitted linear trend lines before and after the Phase I intervention began in the w/c 3rd February 2019. To assess whether differences visible in this graph are statistically significant between intervention and control stores, we will use a difference-in-difference approach, calculating the mean difference in sales (units or £) at each week between intervention and control group, and testing whether this time series of differences changed after intervention time using a linear regression model. We will use a Chow-type test for any difference (in either intercept or slope, or both) after vs. before, and Newey-West standard errors with lag 2 to allow for autocorrelation in the time series. We will use the same approach to evaluate the Phase II intervention but only using data from the 20 intervention stores (12 Phase I and eight Phase II stores) from the 3rd of February 2019 to 1st December 2019; with fitted linear trend lines before and after the Phase II intervention began in the week commencing 28th July 2019.

Sensitivity analysis
If time trends appear to differ between the intervention and control stores, we may define a subgroup of stores which we consider to have comparable sales time trends, and we will perform a re-analysis of the main outcomes.

Sensitivity analyses of the ITS models will test the effect of controlling for seasonality.

Annex A: Specification of pre-intervention exploratory dataset, main analysis dataset, and list of store characteristic variables

Sales data

Each row of the dataset to contain data for one week for a single store

Variables:
Store ID (unique store number)
Store name
Store group (store ID of intervention/test store)
Store category (intervention/control)
Year (2017/2018/2019)
Week (1-52)
Sales (number of units) of mince
Sales (number of units) of burgers
Sales (number of units) of meatballs
Sales (number of units) of sausages
Sales (number of units) of meat-free alternative mince
Sales (number of units) of meat-free alternative burgers[1]
Sales (number of units) of meat-free alternative meatballs
Sales (number of units) of meat-free alternative sausages
Sales (number of units) of other meat-free items
Sales (number of units) of fish
Sales (number of units) of non-dairy milk
Sales (£) of mince
Sales (£) of burgers
Sales (£) of meatballs
Sales (£) of sausages
Sales (£) of meat-free alternative mince
Sales (£) of meat-free alternative burgers
Sales (£) of meat-free alternative meatballs
Sales (£) of meat-free alternative sausages
Sales (£) of other meat-free items
Sales (£) of fish
Sales (£) of non-dairy milk

[1] This category will include ‘veggie fish burgers’ and meat-free steak alternatives
