# Supplementary material for: Estimating the effect of moving meat-free products to the meat aisle on sales of meat and meat-free products: A non-randomised controlled intervention study in a large UK supermarket chain
Source: PLoS Med. 2021 Jul 15;18(7):e1003715. doi: 10.1371/journal.pmed.1003715 (PMC8321099; doi:10.1371/journal.pmed.1003715)
Supplement: S3 Appendix — (DOCX) [file pmed.1003715.s003.docx]

### Supplementary Tables and Figures

### Table A. Demographic store characteristics relating to the surrounding population comparing phase I vs phase II intervention stores

|  | Phase I intervention stores  n=12 | | Phase II intervention stores  n=8 | | χ^2^ test |
| --- | --- | --- | --- | --- | --- |
|  | **n** | **%** | **n** | **%** | P value |
| *Age* |  |  |  |  |  |
| Older | 7 | 58 | 4 | 50 | 0·714 |
| Younger | 5 | 42 | 4 | 50 |  |
| *Affluence* |  |  |  |  |  |
| Less than average | 1 | 8 | 1 | 13 | 0·535 |
| Average | 5 | 42 | 5 | 63 |  |
| More than average | 6 | 50 | 2 | 25 |  |
| *Ethnicity* |  |  |  |  |  |
| White | 6 | 50 | 5 | 63 | 0·582 |
| Asian | 6 | 50 | 3 | 38 |  |
| Other | 0 | 0 | 0 | 0 |  |
| *Area density* |  |  |  |  |  |
| Less urban | 9 | 75 | 3 | 38 | 0·175 |
| Average urban | 3 | 25 | 4 | 50 |  |
| More urban | 0 | 0 | 1 | 13 |  |

### Fig A. Trends in average weekly sales (unit sales) of meat and meat-free products prior to the intervention periods

### Table B. Average weekly sales (units and £) for meat and meat-free products in all intervention and control stores over the pre-intervention baseline and January periods

| *Average weekly sales* | Total stores  n=108 | | Intervention stores  n=20 | | Control stores  n=88 | | Student's  t test |  |
| --- | --- | --- | --- | --- | --- | --- | --- | --- |
| *Baseline period** | **Mean** | **SD** | **Mean** | **SD** | **Mean** | **SD** | **P value** |  |
| Meat (units) | 2396·5 | 1459·5 | 2286·4 | 1305·6 | 2421·5 | 1498·0 | 0·355 |  |
| Meat-free (units) | 173·4 | 112·4 | 172·8 | 117·0 | 173·6 | 112·1 | 0·489 |  |
| Meat (£) | 6525·9 | 4169·3 | 6218·4 | 3741·1 | 6595·8 | 4277·4 | 0·358 |  |
| Meat-free (£) | 414·2 | 276·4 | 410·2 | 286·7 | 415·1 | 275·7 | 0·472 |  |
| *January 2018* | **Mean** | **SD** | **Mean** | **SD** | **Mean** | **SD** | **P value** |  |
| Meat (units) | 2542·0 | 1518·5 | 2385·1 | 1335·2 | 2577·6 | 1561·9 | 0·306 |  |
| Meat-free (units) | 203·3 | 133·5 | 205·3 | 147·7 | 202·8 | 130·9 | 0·530 |  |
| Meat (£) | 7383·7 | 4631·4 | 6924·6 | 4067·5 | 7488·0 | 4765·3 | 0·313 |  |
| Meat-free (£) | 488·9 | 325·9 | 492·2 | 359·9 | 488·1 | 319·9 | 0·520 |  |
| *January 2019* | **Mean** | **SD** | **Mean** | **SD** | **Mean** | **SD** | **P value** |  |
| Meat (units) | 2357·1 | 1446·5 | 2243·2 | 1272·9 | 2383·0 | 1488·5 | 0·349 |  |
| Meat-free (units) | 229·0 | 157·2 | 242·8 | 158·9 | 225·9 | 157·6 | 0·667 |  |
| Meat (£) | 6702·0 | 4314·7 | 6383·1 | 3826·5 | 6774·5 | 4435·1 | 0·358 |  |
| Meat-free (£) | 527·9 | 367·5 | 558·4 | 373·2 | 520·9 | 368·0 | 0·659 |  |
| * Baseline period refers to w/c 2 September 2018 to w/c 18 November 2018 | | | | | | | | |

### Table C. Average baseline sales (units and £) per store per week of meat and meat-free products, % change and comparison of changes between intervention and control stores over the phase I intervention period

|  | **Intervention stores n=20** | | | | | **Control stores n=88** | | | | |  | | | | |
| --- | --- | --- | --- | --- | --- | --- | --- | --- | --- | --- | --- | --- | --- | --- | --- |
|  | **Baseline Period** | | **Phase I Period** | |  | **Baseline Period** | | **Phase I Period** | |  | **Comparison intervention vs control stores** | | | | |
| ***Sales (units)*** | **Mean** | **SD** | **Mean** | **SD** | **% change** | **Mean** | **SD** | **Mean** | **SD** | **% change** | **IRR** | **95%CI** | | **P value** |  |
| **Meat products** | 2289·4 | 1301·9 | 2159·4 | 1229·9 | -6% | 2421·5 | 1498·0 | 2301·7 | 1448·0 | -5% | 1·01 | 0·95 | 1·07 | 0·813 |  |
| Mince | 1269·7 | 736·5 | 1149·8 | 669·0 | -9% | 1348·5 | 850·8 | 1224·3 | 781·8 | -9% | 1·02 | 0·95 | 1·08 | 0·634 |  |
| Burger | 214·5 | 123·0 | 230·1 | 144·7 | 7% | 220·3 | 145·3 | 248·5 | 165·7 | 13% | 0·94 | 0·84 | 1·05 | 0·254 |  |
| Meatballs | 147·0 | 106·8 | 137·5 | 96·6 | -6% | 147·6 | 111·5 | 142·0 | 108·4 | -4% | 0·98 | 0·88 | 1·09 | 0·692 |  |
| Sausages | 658·2 | 352·0 | 641·9 | 336·8 | -2% | 705·0 | 419·4 | 687·0 | 413·6 | -3% | 1·03 | 0·97 | 1·09 | 0·374 |  |
| **Meat-free products** | 173·0 | 116·8 | 226·9 | 113·8 | 31% | 173·6 | 112·1 | 183·6 | 129·2 | 6% | 1·43 | 1·30 | 1·57 | 0·000 |  |
| Mince meat-free | 23·7 | 20·7 | 26·7 | 16·1 | 13% | 24·7 | 19·0 | 23·2 | 16·9 | -6% | 1·50 | 1·13 | 1·99 | 0·005 |  |
| Burger meat-free | 30·1 | 23·9 | 51·0 | 20·0 | 69% | 31·3 | 22·6 | 31·1 | 27·2 | -1% | 2·47 | 1·93 | 3·17 | 0·000 |  |
| Meatballs meat-free | 17·1 | 9·7 | 14·3 | 8·6 | -16% | 15·8 | 8·9 | 14·4 | 8·0 | -9% | 0·91 | 0·68 | 1·21 | 0·507 |  |
| Sausages meat-free | 102·0 | 66·3 | 134·9 | 72·5 | 32% | 101·7 | 65·7 | 114·9 | 81·4 | 13% | 1·33 | 1·22 | 1·45 | 0·000 |  |
| ***Sales (£)*** | **Mean** | **SD** | **Mean** | **SD** | **% change** | **Mean** | **SD** | **Mean** | **SD** | **% change** | **β** | **95%CI** | | **P value** |  |
| **Meat products** | 6226·0 | 3731·9 | 5948·9 | 3587·4 | -4% | 6595·8 | 4277·4 | 6362·9 | 4209·3 | -4% | -35·66 | -120·22 | 48·90 | 0·409 |  |
| Mince | 4029·8 | 2437·1 | 3711·1 | 2266·4 | -8% | 4283·5 | 2808·6 | 3959·1 | 2645·0 | -8% | 2·19 | -50·92 | 55·29 | 0·936 |  |
| Burger | 550·5 | 326·9 | 630·3 | 402·6 | 14% | 565·0 | 384·5 | 686·6 | 474·0 | 22% | -32·30 | -94·19 | 29·58 | 0·306 |  |
| Meatballs | 345·2 | 265·1 | 334·8 | 250·8 | -3% | 345·7 | 273·7 | 343·0 | 275·1 | -1% | -7·27 | -14·05 | -0·49 | 0·036 |  |
| Sausages | 1300·5 | 730·2 | 1272·7 | 701·0 | -2% | 1401·6 | 873·8 | 1374·2 | 859·5 | -2% | -0·65 | -29·92 | 28·63 | 0·965 |  |
| **Meat-free products** | 410·7 | 286·2 | 525·6 | 284·1 | 28% | 415·1 | 275·7 | 436·7 | 309·9 | 5% | 97·63 | 86·74 | 108·51 | 0·000 |  |
| Mince meat-free | 65·9 | 58·8 | 71·0 | 45·5 | 8% | 70·1 | 55·3 | 64·9 | 47·2 | -7% | 9·18 | 6·54 | 11·83 | 0·000 |  |
| Burger meat-free | 60·8 | 53·3 | 110·8 | 50·2 | 82% | 64·1 | 50·9 | 69·2 | 62·1 | 8% | 43·88 | 39·78 | 47·99 | 0·000 |  |
| Meatballs meat-free | 39·6 | 22·5 | 34·7 | 21·5 | -12% | 36·6 | 20·6 | 35·5 | 19·7 | -3% | -2·78 | -4·46 | -1·11 | 0·001 |  |
| Sausages meat-free | 244·4 | 161·1 | 309·2 | 175·1 | 26% | 244·3 | 158·8 | 267·1 | 191·3 | 9% | 47·19 | 40·26 | 54·13 | 0·000 |  |
| *Changes in intervention vs control stores were compared using hierarchical negative binomial models (units) or normal mixed models (£), with fixed effect adjustment for store affluence, store age group, store ethnicity, store area and average units sold per week in 12-week pre-intervention baseline period (w/c 2 September 2018 to w/c 18 November 2018) and a random effects term for matching group | | | | | | | | | | | | | | | |

### Table D. Average baseline sales (units and £) per store per week of other products, % uplift and comparison of changes between intervention and control stores over the phase I intervention period

|  | **Intervention stores n=20** | | | | | **Control stores n=88** | | | | |  | | | |  |
| --- | --- | --- | --- | --- | --- | --- | --- | --- | --- | --- | --- | --- | --- | --- | --- |
|  | **Baseline Period** | | **Phase I Period** | |  | **Baseline Period** | | **Phase I Period** | |  | **Comparison intervention**  **vs control stores** | | | |  |
| ***Sales (units)*** | **Mean** | **SD** | **Mean** | **SD** | **%**  **change** | **Mean** | **SD** | **Mean** | **SD** | **%**  **change** | **IRR** | **95%CI** | | **P value** | |
| Other meat-free products | 425·9 | 298·9 | 564·6 | 385·2 | 33% | 405·6 | 245·3 | 506·6 | 330·7 | 25% | 1·07 | 0·98 | 1·16 | 0·122 | |
| Fish | 3220·4 | 1738·9 | 3311·9 | 1782·6 | 3% | 3215·5 | 1946·0 | 3349·0 | 2030·5 | 4% | 1·03 | 0·98 | 1·08 | 0·214 | |
| Non-dairy milk | 472·8 | 314·0 | 494·7 | 318·6 | 5% | 439·0 | 274·8 | 449·5 | 272·2 | 2% | 1·02 | 0·95 | 1·11 | 0·558 | |
| Vegetables | 24914·3 | 13542·5 | 25290·6 | 13607·6 | 2% | 25145·1 | 13883·1 | 25639·7 | 14361·3 | 2% | 1·01 | 0·96 | 1·06 | 0·713 | |
| Fruit | 16955·8 | 9934·6 | 17694·7 | 10146·0 | 4% | 16544·4 | 9207·4 | 17347·4 | 9741·0 | 5% | 0·99 | 0·95 | 1·04 | 0·741 | |
| Personal Care | 31120·1 | 19122·6 | 29859·9 | 18154·7 | -4% | 31895·7 | 19674·1 | 30587·0 | 18870·6 | -4% | 1·01 | 0·95 | 1·08 | 0·683 | |
| ***Sales (£)*** | **Mean** | **SD** | **Mean** | **SD** | **%**  **change** | **Mean** | **SD** | **Mean** | **SD** | **%**  **change** | **β** | **95%CI** | | **P value** | |
| Other meat-free products | 916·4 | 658·5 | 1115·9 | 796·3 | 22% | 876·1 | 542·1 | 1007·9 | 675·4 | 15% | 71·86 | 57·07 | 86·66 | 0·000 | |
| Fish | 10766·6 | 6257·5 | 11386·4 | 6552·9 | 6% | 10643·5 | 6867·8 | 11420·7 | 7352·8 | 7% | -135·51 | -280·53 | 9·51 | 0·067 | |
| Non-dairy milk | 651·2 | 440·3 | 688·7 | 449·1 | 6% | 603·6 | 384·6 | 629·5 | 390·0 | 4% | 16·95 | 4·27 | 29·63 | 0·009 | |
| Vegetables | 23596·7 | 13343·1 | 24723·1 | 13858·3 | 5% | 23742·6 | 13585·1 | 25033·4 | 14483·7 | 5% | -125·77 | -436·49 | 184·94 | 0·428 | |
| Fruit | 23089·8 | 14788·1 | 24698·6 | 15367·7 | 7% | 22291·8 | 13413·1 | 23922·8 | 14348·6 | 7% | -0·40 | -214·84 | 214·04 | 0·997 | |
| Personal Care | 73963·1 | 47405·5 | 71182·7 | 45474·3 | -4% | 75603·0 | 48706·4 | 72798·9 | 47170·1 | -4% | 138·22 | -355·30 | 631·73 | 0·583 | |
| *Changes in intervention vs control stores were compared using hierarchical negative binomial models (units) or normal mixed models (£), with fixed effect adjustment for store affluence, store age group, store ethnicity, store area and average units sold per week in 12-week pre-intervention baseline period (w/c 2 September 2018 to w/c 18 November 2018) and a random effects term for matching group | | | | | | | | | | | | | | |  |

### Table E. Sensitivity analysis comparing changes in the primary outcome (unit sales) between intervention and control stores over the phase I intervention period adjusting for an alternative baseline period (w/c 4 February 2018 to w/c 22 April 2018) which matches the intervention period (w/c 3 February 2019 to w/c 21 April 2019)

|  |  | | | | |
| --- | --- | --- | --- | --- | --- |
|  | **Comparison intervention vs control stores** | | | | |
| ***Sales (units)*** | **IRR** | **95%CI** | | **P value** |  |
| **Meat products** | 1·03 | 0·97 | 1·10 | 0·327 |  |
| Mince | 1·05 | 0·98 | 1·12 | 0·178 |  |
| Burger | 0·97 | 0·87 | 1·08 | 0·606 |  |
| Meatballs | 1·04 | 0·93 | 1·16 | 0·544 |  |
| Sausages | 1·03 | 0·97 | 1·10 | 0·334 |  |
| **Meat-free products** | 1·41 | 1·27 | 1·56 | <0·0001 |  |
| Mince meat-free | 1·46 | 1·09 | 1·95 | 0·011 |  |
| Burger meat-free | 2·43 | 1·85 | 3·18 | <0·0001 |  |
| Meatballs meat-free | 1·01 | 0·69 | 1·49 | 0·946 |  |
| Sausages meat-free | 1·31 | 1·20 | 1·44 | <0·0001 |  |

*Changes in intervention vs control stores were compared using hierarchical negative binomial models (units) or normal mixed models (£), with fixed effect adjustment for store affluence, store age group, store ethnicity, store area and average units sold per week in 12-week pre-intervention baseline period (w/c 4 February 2018 to w/c 22 April 2018) and a random effects term for matching group

**Fig B. Interrupted time series analysis showing level and trend changes in sales (£) of meat and meat-free products before and after phase I intervention (week commencing 3rd February 2019) in intervention stores (green n=20) and control stores (red n=88)**


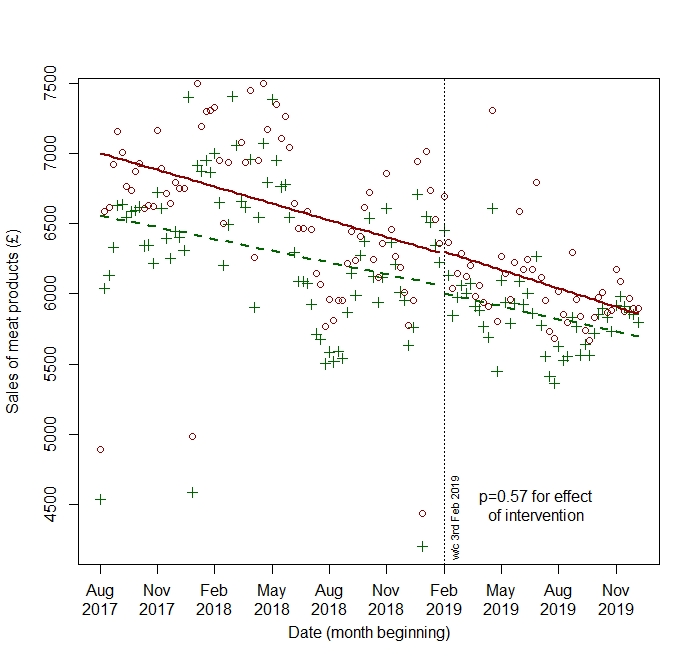

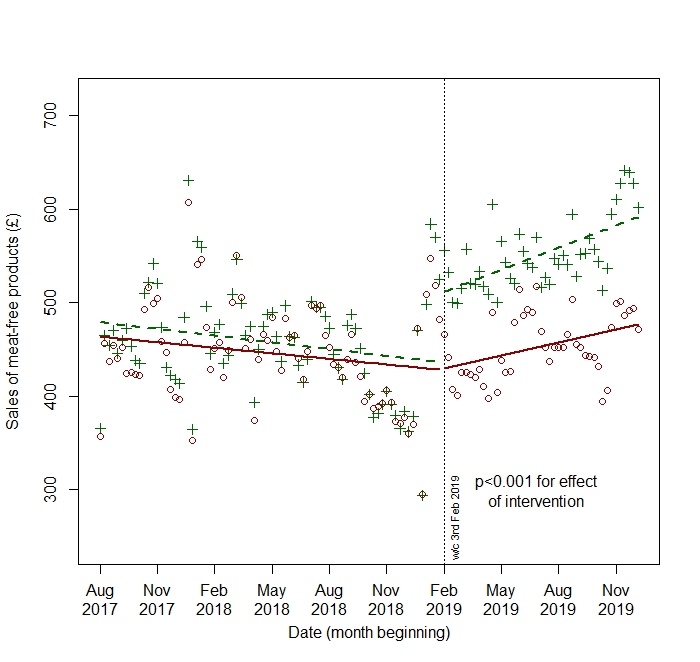


**Fig C. Interrupted time series analysis showing level and trend changes in sales (difference-in-difference, expressed in units and £) of meat and meat-free products before and after phase I intervention (week commencing 3rd February 2019) between intervention stores and control stores.**


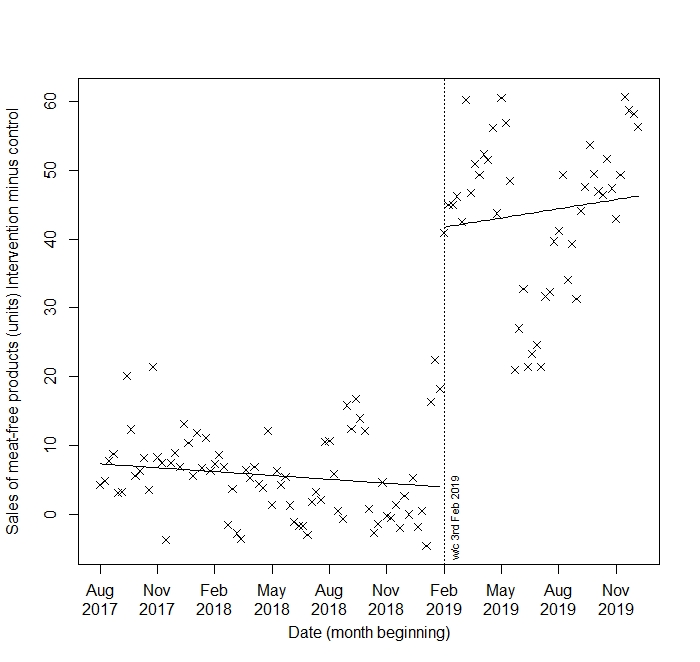

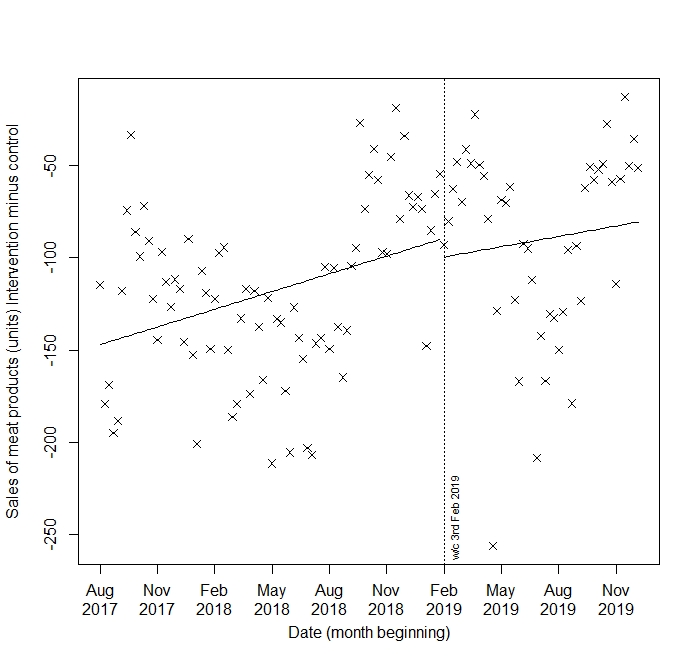

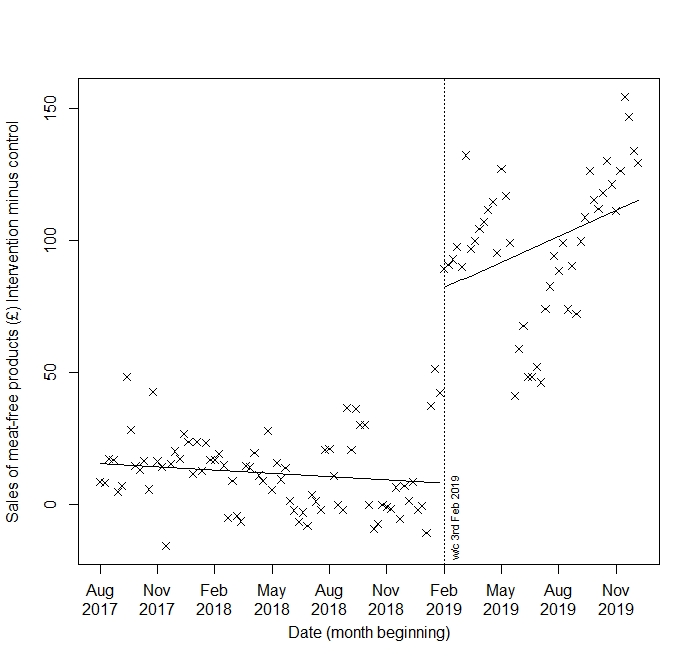

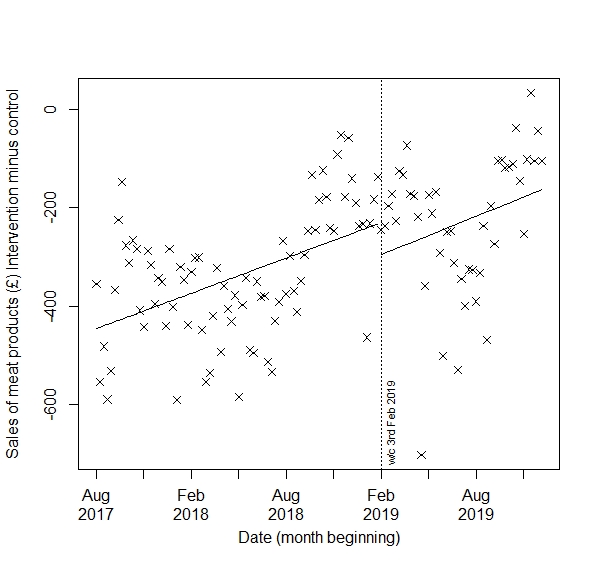


### Fig D. Sensitivity analysis on the interrupted time series analysis showing level and trend changes in sales (difference-in-difference, expressed in Units) of meat and meat-free products before and after phase I intervention (week commencing 3rd February 2019) in intervention stores (green n=20) and control stores (red n=88), adjusting for seasonality

w/c 3^rd^ Feb 2019

w/c 3^rd^ Feb 2019

P<0.001 for effect of intervention

P=0.07 for effect of intervention

###

### Table F. Average baseline sales (units and £) per store per week of meat and meat-free products, % change and comparison of changes between intervention and control stores over the phase I and phase II intervention periods

|  | **Intervention Phase I stores n=12** | | | | | **Intervention Phase II stores n=8** | | | | |  | | | |
| --- | --- | --- | --- | --- | --- | --- | --- | --- | --- | --- | --- | --- | --- | --- |
|  | **Phase I Period** | | **Phase II Period** | |  | **Phase I Period** | | **Phase II Period** | |  | **Comparison phase I and phase II stores** | | | |
| ***Sales (units)*** | **Mean** | **SD** | **Mean** | **SD** | **% change** | **Mean** | **SD** | **Mean** | **SD** | **% change** | **IRR** | **95%CI** | | **P value** |
| **Meat products** | 2401·9 | 1338·3 | 2427·7 | 1322·2 | 1% | 1795·7 | 1019·5 | 1814·4 | 985·6 | 1% | 0·97 | 0·89 | 1·06 | 0·476 |
| Mince | 1308·1 | 731·2 | 1320·6 | 715·2 | 1% | 912·3 | 517·1 | 940·7 | 534·4 | 3% | 0·94 | 0·86 | 1·03 | 0·164 |
| Burger | 249·9 | 158·6 | 266·8 | 156·3 | 7% | 200·5 | 125·2 | 199·9 | 106·4 | 0% | 1·03 | 0·92 | 1·16 | 0·596 |
| Meatballs | 163·8 | 106·3 | 182·0 | 116·3 | 11% | 98·2 | 68·2 | 109·9 | 67·7 | 12% | 0·96 | 0·86 | 1·08 | 0·536 |
| Sausages | 680·0 | 352·5 | 658·3 | 341·8 | -3% | 584·8 | 326·3 | 563·9 | 293·2 | -4% | 0·99 | 0·90 | 1·10 | 0·908 |
| **Meat-free products** | 245·4 | 123·2 | 237·7 | 129·5 | -3% | 199·1 | 99·1 | 221·3 | 126·2 | 11% | 1·24 | 1·05 | 1·48 | 0·014 |
| Mince meat-free | 30·0 | 18·7 | 33·4 | 19·8 | 11% | 21·7 | 10·3 | 29·5 | 15·9 | 36% | 1·26 | 0·95 | 1·68 | 0·112 |
| Burger meat-free | 52·1 | 20·2 | 41·2 | 19·8 | -21% | 49·3 | 21·0 | 36·4 | 18·0 | -26% | 0·98 | 0·81 | 1·18 | 0·838 |
| Meatballs meat-free | 15·9 | 6·5 | 19·9 | 11·2 | 26% | 11·9 | 11·0 | 17·6 | 12·0 | 47% | 1·31 | 0·88 | 1·94 | 0·187 |
| Sausages meat-free | 147·4 | 79·4 | 143·1 | 80·3 | -3% | 116·1 | 60·5 | 137·9 | 82·2 | 19% | 1·27 | 1·02 | 1·57 | 0·031 |
| ***Sales (£)*** | **Mean** | **SD** | **Mean** | **SD** | **% change** | **Mean** | **SD** | **Mean** | **SD** | **% change** | **β** | **95%CI** | | **P value** |
| **Meat products** | 6728·3 | 3942·1 | 6429·4 | 3683·8 | -4% | 4779·7 | 2812·0 | 4621·6 | 2699·6 | -3% | 9·1 | -210·6 | 228·8 | 0·935 |
| Mince | 4260·8 | 2498·2 | 4002·2 | 2279·1 | -6% | 2886·6 | 1685·2 | 2794·9 | 1680·0 | -3% | 5·4 | -135·2 | 146·0 | 0·940 |
| Burger | 691·9 | 444·5 | 734·7 | 437·9 | 6% | 538·0 | 336·5 | 538·8 | 300·6 | 0% | 7·3 | -83·7 | 98·4 | 0·875 |
| Meatballs | 403·2 | 275·6 | 420·8 | 281·8 | 4% | 232·0 | 177·0 | 249·8 | 168·4 | 8% | -4·1 | -23·9 | 15·7 | 0·684 |
| Sausages | 1372·4 | 748·7 | 1271·7 | 700·7 | -7% | 1123·0 | 640·5 | 1038·2 | 578·6 | -8% | 1·4 | -74·7 | 77·5 | 0·971 |
| **Meat-free products** | 571·3 | 306·7 | 570·4 | 325·1 | 0% | 457·2 | 249·7 | 525·6 | 311·9 | 15% | 100·3 | 38·3 | 162·4 | 0·002 |
| Mince meat-free | 80·4 | 53·3 | 92·7 | 56·7 | 15% | 56·9 | 27·9 | 78·9 | 44·0 | 39% | 11·4 | -6·3 | 29·1 | 0·206 |
| Burger meat-free | 113·4 | 50·1 | 92·0 | 48·3 | -19% | 106·9 | 53·5 | 81·4 | 44·5 | -24% | 2·9 | -13·0 | 18·8 | 0·721 |
| Meatballs meat-free | 38·6 | 16·8 | 45·9 | 26·4 | 19% | 28·8 | 27·4 | 40·5 | 27·4 | 40% | 6·9 | -7·3 | 21·0 | 0·340 |
| Sausages meat-free | 338·9 | 190·7 | 339·7 | 197·8 | 0% | 264·6 | 149·4 | 324·7 | 199·7 | 23% | 68·7 | 26·0 | 111·3 | 0·002 |
| *Changes in intervention phase I vs phase II stores were compared using hierarchical negative binomial models (units) or normal mixed models (£), with fixed effect adjustment for store affluence, store age group, store ethnicity, store area and average units sold per week in 12-week phase I period (w/c 3 February 2019 to w/c 28 April 2019) and a random effects term for matching group | | | | | | | | | | | | | | |

### Table G. Average baseline sales (units and £) per store per week of other products, % change and comparison of changes between intervention and control stores over the phase I and phase II intervention periods

|  | **Intervention Phase I stores n=12** | | | | | **Intervention Phase II stores n=8** | | | | |  | | | |  |
| --- | --- | --- | --- | --- | --- | --- | --- | --- | --- | --- | --- | --- | --- | --- | --- |
|  | **Phase I Period** | | **Phase II Period** | |  | **Phase I Period** | | **Phase II Period** | |  | **Comparison phase I and phase II stores** | | | |  |
| ***Sales (units)*** | **Mean** | **SD** | **Mean** | **SD** | **%**  **change** | **Mean** | **SD** | **Mean** | **SD** | **%**  **change** | **IRR** | **95%CI** | | **P value** | |
| Other meat-free products | 596·4 | 426·9 | 687·4 | 447·1 | 15% | 517·0 | 334·8 | 685·4 | 404·7 | 33% | 1·26 | 0·99 | 1·60 | 0·057 | |
| Fish | 3770·7 | 2017·5 | 3699·2 | 1919·3 | -2% | 2623·6 | 1152·0 | 2682·3 | 1159·5 | 2% | 1·03 | 0·92 | 1·16 | 0·628 | |
| Non-dairy milk | 497·9 | 298·9 | 449·3 | 272·3 | -10% | 489·9 | 367·5 | 428·1 | 321·2 | -13% | 0·88 | 0·75 | 1·03 | 0·109 | |
| Vegetables | 27697·9 | 14827·1 | 27431·9 | 14322·8 | -1% | 21679·7 | 11501·7 | 21736·2 | 11907·6 | 0% | 0·96 | 0·87 | 1·05 | 0·328 | |
| Fruit | 19157·9 | 10288·9 | 19432·7 | 10034·3 | 1% | 15499·9 | 10192·4 | 16120·3 | 10941·5 | 4% | 0·92 | 0·82 | 1·02 | 0·123 | |
| Personal Care | 32269·8 | 18776·2 | 32574·6 | 18609·1 | 1% | 26245·1 | 17768·5 | 26761·2 | 18042·1 | 2% | 0·92 | 0·81 | 1·05 | 0·217 | |
| ***Sales (£)*** | **Mean** | **SD** | **Mean** | **SD** | **%**  **change** | **Mean** | **SD** | **Mean** | **SD** | **%**  **change** | **β** | **95%CI** | | **P value** | |
| Other meat-free products | 1193·5 | 888·7 | 1424·3 | 965·5 | 19% | 999·3 | 673·9 | 1426·9 | 874·6 | 43% | 203·8 | 47·1 | 360·5 | 0·011 | |
| Fish | 12941·7 | 7402·3 | 11792·2 | 6559·7 | -9% | 9053·4 | 4481·0 | 8537·1 | 4183·3 | -6% | 48·3 | -335·4 | 432·1 | 0·805 | |
| Non-dairy milk | 695·9 | 424·7 | 643·4 | 395·4 | -8% | 677·9 | 513·7 | 609·9 | 465·7 | -10% | -12·3 | -45·4 | 20·7 | 0·464 | |
| Vegetables | 27100·3 | 14985·1 | 25433·2 | 13721·6 | -6% | 21157·2 | 12008·2 | 20185·4 | 11846·8 | -5% | -101·3 | -1030·8 | 828·1 | 0·831 | |
| Fruit | 26905·2 | 15485·6 | 25684·7 | 14143·0 | -5% | 21388·6 | 15598·7 | 20758·8 | 15802·0 | -3% | 121·1 | -1229·1 | 1471·3 | 0·860 | |
| Personal Care | 77156·1 | 46985·3 | 78695·3 | 47074·2 | 2% | 62222·5 | 44619·2 | 63872·8 | 45493·3 | 3% | -864·6 | -3571·3 | 1842·1 | 0·531 | |
| *Changes in intervention phase I vs phase II stores were compared using hierarchical negative binomial models (units) or normal mixed models (£), with fixed effect adjustment for store affluence, store age group, store ethnicity, store area and average units sold per week in 12-week phase I period (w/c 3 February 2019 to w/c 28 April 2019) and a random effects term for matching group | | | | | | | | | | | | | | | |

### Table H. Results of Phase I (n=20) and Phase II (n=8) in-store fidelity evaluations

Researchers carried out in-store fidelity evaluations of all Phase I and Phase II intervention stores and a sample of control stores, on two unannounced occasions throughout the intervention periods (one week day and one weekend day). These visits aimed to assess whether the intervention was implemented as planned. For the Phase I visits all community researchers were provided with a data collection form and a planogram of the meat-free products. The following details were captured: confirmation the meat-free section had been re-positioned to the meat aisle; whether the meat-free section was located at the end or the middle of the meat aisle and if the latter, what the adjacent product categories were; whether each item in the meat-free planogram was in stock, out of stock (i.e. the product wasn’t available but a product shelf label was visible) or missing (i.e. no product or shelf label); whether any of the meat-free alternatives were on promotion, how many (if any) products on the middle two (eye-level) shelves in the planogram were incorrectly stocked on the higher or lower shelves; and whether any of the meat-free items in the planogram were stocked elsewhere in store (for example the ‘vegetarian section’ where meat-free items are traditionally stocked). Photographs were also taken of the meat-free section to document the products on sale, stock levels, presence and promotion of point of purchase (POS) displays, signposting (presence/absence of a ‘meat alternatives’ header board and section dividers, as well as the overall appearance of the section. For the Phase II visits, community researchers were asked to confirm whether an additional meat-free section had been re-positioned to the meat aisle. They were also asked to take photographs of this section; including close up pictures of each shelf and a wider photo showing the entire meat-free section and the product categories either side. Community researchers visiting the control stores were asked to confirm that none of the meat-free alternatives in the meat-free planogram were located in the meat aisle.

| Phase I Intervention Component | Number of stores (%) |
| --- | --- |
| Meat-free section had been re-positioned to the meat aisle | 20 (100%) |
| Meat-free section was located at the end of the meat aisle | 13 (65%) |
| Meat-free section was located in the middle of the meat aisle | 7 (35%) |
| At least 80% of items in the meat-free planogram were in stock | 16 (62%) |
| At least 50% of items in the meat-free planogram were in stock | 20 (100%) |
| Any of the meat-free items in the planogram were out of stock but product shelf label still visible | 20 (100%) |
| Any of the meat-free items in the planogram were missing and no product shelf label was visible | 15 (75%) |
| Any of the meat-free items in the planogram were on promotion | 20 (100%) |
| Any of the meat-free items on the middle two (eye-level) shelves in the planogram were incorrectly stocked on the higher or lower shelves | 4 (20%) |
| Any of the meat-free items in the planogram were stocked elsewhere in store | 6 (30%) |
| At least one point of sale display (‘Plant Power’ or ‘Simple Swaps’) was in the meat-free section | 13 (65%) |
| Phase II Intervention Component |  |
| Second bay of meat-free items was added next to original bay | 7 (87.5%) |
