# Supplementary material for: Estimating the effect of moving meat-free products to the meat aisle on sales of meat and meat-free products: A non-randomised controlled intervention study in a large UK supermarket chain
Source: PLoS Med. 2021 Jul 15;18(7):e1003715. doi: 10.1371/journal.pmed.1003715 (PMC8321099; doi:10.1371/journal.pmed.1003715)
Supplement: Attachment — Submitted filename: Positioning Plos Med 1st revision.docx [file pmed.1003715.s004.docx]

"Estimating the effect of moving meat-free products to the meat aisle on sales of meat and meat-free products: a non-randomised controlled intervention study in a large UK supermarket chain" (PMEDICINE-D-21-00493R1)

Reviewer #1: This non-randomised controlled intervention study aims to evaluate whether prominent positioning of meat-free products in the meat aisle was associated with a change in weekly mean sales of meat and meat-free products.

| Reviewer comments | Authors response | Changes made |
| --- | --- | --- |
| Did the authors undertake any sample size calculations (a priori) to provide an estimate of statistical power for the study of 20 stores with matched controls? | We didn’t conduct a power analysis as indicated in our pre-published protocol. Instead, we followed the conduct and evaluation of a natural experiment where the retailer chose to test the intervention in 20 stores (with a sample of matched control stores) with the view of estimating the effect sizes for a future trial. However the retailer did not continue their plans to conduct a larger trial. We have included a sentence to clarify this in the methods section. | Page 8  “Following the conduct and evaluation of a natural experiment, a power analysis was not conducted and the final number of trial stores with matched control stores was chosen by the retailer” |
| A randomised control design would have been more rigorous and robust, with greater strength of conclusions in terms of inferring causality and minimising the risk of confounding.  However, it is good that a matched-control design has been followed, which goes some way towards understanding what might have happened in the scenario of no intervention. | We agree with this comment. In the analysis of this natural experiment, the matched controlled design (with adjustment for store demographics) is able to estimate the effects of the intervention in a more robust way given the lack of randomisation. Although this method cannot overcome residual confounding completely, it has been widely used in previous analyses of natural experiments. |  |
| Do the 108 stores included represent the wider population of stores for this retail partner? | Yes, the 108 stores are distributed across most regions of the UK and include small, medium and large supermarket sites. We have clarified this in our methods section and added store size samples to Table 1. | Page 6  “The final sample of 108 stores included in this analysis were representative of the retail partner store population, with stores distributed across all UK regions, including small, medium and large supermarket sites.” |
| "Intervention sites were all stores where there was unproductive space in the meat aisle. In other words, the meat sales did not justify the volume of inventory or number of product bays. For this study, 20 stores were selected based on the retailer's estimates of the opportunity cost of removing meat products, as well as operational considerations. The stores were selected to have a broad spread with regard to the following characteristics: 1) affluence (based on top 2 Acorn consumer classification categories[17] within a 10 minute drive, in combination with meat-free sales performance (assessed in the 12 week period ending 25 September 2018)); and 2) store size (large, medium, small)."  Whilst Table 1 shows little difference between the characteristics of the 20 intervention stores and their matched controls, Figure 2 (left) suggests that intervention and control groups had different purchasing behaviours pre-intervention?  Figure 2 also shows that control stores saw a slope change, despite no intervention - what might be the reasons for this? | One of the limitations of a natural experiment is that the stores were not randomised. However, as recognised by the reviewer, we didn’t find significant differences in demographic characteristics between intervention and control stores (Table 1). We cannot exclude other differences but we have no evidence of them.  Figure 2 shows the ITS analysis in which sales of meat-free items are completely overlapping and show the exact same trend pre-intervention.  Sales of meat products in Figure 2 showed small absolute differences but a very similar trend pre-intervention. We tested these differences in sales (£ and units) between intervention and control stores over the baseline period (w/c 2 September 2018 to w/c 18 November 2018) and January 2018 and 2019 periods which showed no statistically significant differences (see Supplementary table 2). Therefore we are as confident as we can be that the selected stores were fairly comparable with regards to demographic characteristics, absolute sales of meat and meat-free products and trends in sales pre-intervention.  The observed slope change in control stores may be due to the fact that more people are choosing meat-free products. This is also reflected in Suppl table 2 which showed that sales of meat-free are higher in January 2019 vs 2018 in both intervention and control stores. |  |
| "The primary outcome analysis used a hierarchical negative binomial model to compare changes in weekly sales (units) of meat products sold in intervention vs control stores during the main intervention period (Phase I-February 2019-April 2019). Interrupted time series analysis was also used to evaluate the effects of the phase I intervention."  The modelling technique applied is appropriate for the data and research question.  Can the authors please clarify whether they compared weekly sales, or changes in weekly sales? | The hierarchical negative binomial/mixed models used weekly sales of meat and meat-free products as the dependent variable.  These models also included adjustment for baseline sales, therefore the model effectively estimates the change in weekly sales over the intervention period against the baseline period, for the intervention stores compared to control stores. |  |
| Seasonality is almost certainly at play in this data, as acknowledged by the authors.  It is noted that: "The preintervention baseline period and the intervention period were defined a priori with the retail partner to avoid months where meat and meat-free purchases are known to be atypical (e.g. Christmas, January, summer)." This is good practice, but does not explore whether baseline months might be different to Phase I and Phase II intervention months, due to reasons other than different products in the meat aisle (such as seasonality).  The authors go on to say that: "In sensitivity analyses we tested the effect of controlling for seasonality and changes in purchases over January." Can the authors please present these sensitivity analyses in the supplementary material? | We appreciate the reviewer comment and agree seasonality is important to be considered here, although arguably less relevant for meat and meat-free items compared to fruits or vegetables for which there are much larger variations across the year. The other important consideration is that, if seasonality appears to be similar between intervention and control stores, then this should not be a major problem in this analysis. We tried to explore differences in seasonality between intervention and control stores and included a trend analysis in Supplementary Figure 1 where we show that sales of meat and meat-free products pre-intervention follow very similar trends across the months in the intervention and control stores.  Secondly, we performed a sensitivity analysis of the ITS models which controlled for seasonal changes which showed consistent results (results included now in supplementary Figure 4). We have mentioned this in the results section too. | Supplementary Figure 4  Results Page 15 |
| Given the longitudinal data available prior to intervention, this seasonality effect across a complete year could be explored? ("Data on store-level weekly sales (units purchased and £) of meat and meat-free products were obtained for August 2017 to December 2019 (123 weeks) for 108 stores (20 intervention and 88 matched control stores), resulting in 13,284 total store-weeks of data").  An alternative approach might be to compare the same months as a sensitivity analysis? i.e. Feb-Apr 2018 compared to Feb-Apr 2019? | See above our comment regarding the sensitivity analysis adjusted for the effect of seasonal changes in the ITS models.  We have also included a new sensitivity analysis on the hierarchical mixed models adjusting for an alternative baseline period (w/c 4 February 2018 to w/c 22 April 2018) which matches the intervention period (w/c 3 Feb 2019 to w/c 21 April 2019); and this new analysis has generally showed consistent results. This is also discussed in the results section. | Supplementary Table 5  Results page 13 |
| The authors have suitably included covariates in their analysis in an attempt to account for various potential sources of confounding that may be at play:  "These measures were included as covariates in the analyses: affluence coded as average, >average, <average; age group coded as older vs younger; ethnicity coded as White, Asian, Other; area density coded as urban, more urban, less urban. " | That’s correct we controlled for any of the available confounders (store demographics and baseline sales) despite the lack of significant differences between intervention and control stores, therefore we are confident we have removed confounding as much as possible. |  |
| "To assess whether differences visible in this graph were statistically significant between intervention and control stores, we used a difference-in-difference approach, calculating the mean difference in sales (£) at each week between intervention and control group, and testing whether this time series of differences changed after intervention time using a linear regression model. We used a Chow-type test for any difference (in either intercept or slope, or both) after vs. before, and used Newey-West standard errors with lag to allow for autocorrelation in the time series"  The authors have calculated aggregate differences between control and intervention groups, and compared the linear regressions before vs. after intervention.  This is a novel approach which attempts to draw the most from the data at hand. However, it is not necessarily the most intuitive methodology, and may be difficult to interpret given the other possible differences between control and intervention groups.  Did the authors also consider testing for differences in linear regressions for intervention groups and control groups, before vs. after (i.e. comparing intervention to intervention, or control to control, before and after intervention)? This additional analysis may help to provide some depth and improved understanding as to what is changing over time, and how. | We appreciate this comment but we have previously commented on the lack of significant differences between intervention vs control stores in terms of baseline sales and trends pre-intervention (Suppl Table 2; Suppl Figure 1).  The difference in difference approach is widely used as it allows to further account for small time-related fluctuations and reduces the effect of absolute differences between the intervention and control stores in the estimations (which are not significant in any case). We have included a new set of graphs in Supplementary Figure 3 showing ITS difference-in-difference models.  Regarding the suggestion of testing for differences before vs after, our hierarchical mixed/negative binomial models are exactly aiming to do this. These models are adjusted for baseline sales (over the period of Sept-Nov 2018 to avoid the Christmas period as explained above), therefore we test for the differences in sales over the intervention period between intervention vs control stores. This approach is effectively testing for differences before/after between intervention and control stores. | Supplementary Figure 3 |
| "Eight of the 20 stores enhanced the intervention from August 2019 onwards (Phase II intervention) by moving the entire meat-free section into the meat aisle, which was evaluated following the same analytical methods."  Thanks to Phase II of the study, the authors now have access to a cohort of stores with a) no intervention, b) some intervention and c) full intervention. Did they consider running analyses across all three of these groups in an attempt to understand if a dose-response relationship could be measured? | We believe our results support the idea that there could potentially be a dose-response relationship. We investigated the effect of the Phase II intervention in 2 possible ways. Firstly, we run the same hierarchical mixed/negative binomial models where we compare phase II vs phase I stores adjusting for sales over the phase I period (Suppl Table 6). Secondly, the ITS analysis supports the results observed in the mixed models which showed a significant increase in meat-free items over the phase II period. These 2 approaches allowed us to detect the effect of phase II over and above what phase I achieved, and these changes would likely be significant had we compared against control stores. However we are reluctant to perform any more explorations of the phase II intervention because it was not part of the pre-planned intervention and was only carried out in a very small sample of stores (n=8). |  |
| Similar to the non-randomisation for the original 20 stores which may cause a bias in the data, there may be some issue regarding the selection of these 8 stores: "These eight stores were selected by the retailer based on sufficient meat-free products in stock." What might this imply about the sample of 8 stores with enhanced intervention? Is there a bias here, that might suggest the enhanced intervention was more likely to be effective? | As stated in our manuscript, the phase II had not been pre-planned and the choice of the 8 phase II stores was entirely made by the retailer based on the available space and capacity to stock a full bay of meat-free products. But we attempted to understand if there were any differences that could explain the results. Firstly we showed there were no significant differences in demographic characteristics (Suppl Table 1). Secondly, our mixed models in Suppl Table 6 are adjusted for sales over the phase I period to account for absolute differences in sales between the 8 stores compared to the 12 stores that continued with the phase I intervention. Our difference-in-difference approach in the ITS model also accounts for differences in absolute sales before the phase II intervention was implemented. Therefore we are confident our results are robust to any potential bias that may have been introduced by the choice of the phase II stores. |  |
| Figure 3 shows differences between the Phase I and enhanced intervention group before Phase II started. The Phase I stores saw a step and slope change, despite no additional intervention. Why might this be? | As pointed out by the reviewer, Figure 3 ITS showed a reduction (step change) in meat-free sales in the Phase I stores despite no changes in the intervention, followed by a slope change. However, suppl table 6 showed average sales over the entire phase II period when there was a small decrease in meat-free sales (-3% units; 0% £ sales) in the intervention stores which remained in phase I intervention, suggesting there were very minor changes compared to the phase I period. But as previously said, we can’t really explain phase II in much more detail as this intervention wasn’t planned a priori and any conclusions need to be interpreted in an exploratory way. |  |
| "A small number of control stores was initially matched to more than one intervention store. In these instances, we manually selected only one control per intervention store. Therefore, of the 20 intervention stores, nine had five matched controls, ten had four matched controls, and one had three matched control stores. "  Can the authors please clarify - each control store has been uniquely matched to one intervention store? (i.e. the 88 controls refer to 88 distinct stores)? | We have clarified in the manuscript that each control store was uniquely matched to each intervention store. | Methods Page 6 |
| "A preregistered protocol (<https://osf.io/qmz3a/>) was completed and fully available from April 2019 before obtaining data for analyses. A more detailed statistical analysis plan was completed in January 2020 after data cleaning but before data analysis. "  Can the authors please provide these within the supplementary material? | We have provided both protocol and SAP as supplementary material |  |
| The text of the manuscript suggests that one particular retail partner or supermarket chain is included in the analysis. It is appreciated that possibly the supermarket chain cannot be named, however, can the authors please discuss how the results seem here may or may not be applicable to other chains? (different market and clientele, pricing structures etc. may affect generalisation of the study findings?). | Our retail partner for this research is a national supermarket chain with one of the largest shares of the grocery sector. Its offerings are targeted to a general and broad customer base rather, i.e. not focussed on premium or discount customers or specific diets. Therefore, we believe it was a good choice for research that would be applicable to the general population. We have added a sentence about this to the discussion section. | Discussion Page 22  “Our retail partner is a national supermarket chain with one of the largest shares of the grocery sector, and its offerings are targeted to a general and broad customer base, which increases the generalisability of the findings to the broader UK population” |
| Of note, the title of the Supplementary Material document names the retail partner.  Given the authors have (presumably deliberately) not specified this within the manuscript, should this title be changed for commercial confidentiality reasons? | Thanks for noting this! We have now anonymised our files |  |
| Supplementary Figure 1 shows identical weekly sales (where the lines completely overlap)- is this correct? | We believe the reviewer is referring to the lines representing meat-free sales in Suppl Figure 1. Sales in intervention vs control stores in this trend figure look to overlap completely which is correct, but in reality there are some small differences which can be appreciated in Figure 2 meat-free sales panel (see sales before intervention line). |  |

Reviewer #2: I congratulate the team for the design, delivery and analysis of this impressive experiment - this is exactly the sort of information that is needed to help understand the potential impact of food industry-led interventions to change dietary practices in the UK. This has clearly been a hugely difficult study to conduct given what seems to have been quite careful "management" of the study by your industry partner and a change to the protocol mid-way through the intervention. My comments below relate largely to the accurate and appropriate reporting of your study.

| Reviewer comments | Authors response | Changes made |
| --- | --- | --- |
| 1. The very helpful online protocol calls this a "pilot non-randomised before and after study". Indeed, the protocol states that one of the secondary aims of your "pilot" study is to "to obtain data needed to design and conduct a larger, more definitive in-store intervention study". Is this the case, in which should this study be better re-titled to acknowledge its pilot nature? It's noteworthy for example that there is no sample size calculation in the manuscript (you justify this in the protocol by stating this is a pilot study). | We thank the reviewer for their positive feedback and have tried to clarify all their concerns where possible.  As the reviewer noted, this study was initially conceived with the retailer as a pilot with the view to conduct a larger study afterwards, and this was reflected in the pre-registered protocol. Therefore a power calculation was not performed and the retailer chose the number of stores to roll out the intervention. However, the retailer changed these plans once the study had started and decided to implement the phase II intervention (intensify the intervention in a smaller sample of stores), instead of rolling out a larger trial.  We have added a sentence to the Methods to reflect this. We have also included in the submission a revised Protocol and SAP indicating the deviation. | Methods page 8:  “Following the conduct and evaluation of a natural experiment, a power analysis was not conducted and the final number of trial stores with matched control stores was chosen by the retailer.” |
| 2. The online protocol relates to the study as originally planned - and this does not entirely reflect what actually happened (i.e. Phase 2). If this was an RCT then this would of course have been a major protocol change and the protocol would have need to be update. I realise that this was not an RCT but I wonder if it would have been appropriate to update your protocol to reflect what actually happened. | Yes, this is a fair point. As is the case with these collaborations with retailers, we are not in control of the implementation process. Our agreed upon protocol reflected only Phase 1 and during the trial the retailer decided upon itself to extend the intervention in 8 stores. We seized upon this opportunity to analyse what we deemed to be a Phase 2 but had not updated the protocol accordingly. We have included now the original protocol which includes an explanation about the Phase II intervention. We are learning as we go with these ‘real-world’ evaluations and this is a reminder to us to update our protocols as we go in future. |  |
| 3. The description of the intervention(s) could be improved to help the reader. There is currently insufficient distinction between the description of Phase 1 "to their own meat-free bay in the meat aisle (l.122-123) and Phase 2 "moving the entire bay of meat-free products in the mat aisle" (l.136-137). It's not clear what "the entire bay" consists of and how that is different from "their own meat-free bay". "The meat-free bay was the last bay in the meat aisle…" surely depends on the direction in which you access the aisle - maybe "end bay" would be an alternative? It's not clear what happened in the stores between April 2019 and July 2019 (i.e. l.132-139 needs to be re-written). | We have rewritten this paragraph as suggested to clarify the interventions over the phase I and phase II periods. | Methods page 7  Phase I  The intervention was implemented in 20 stores on the week commencing 27th January 2019, for 12 weeks. In the control stores, meat-free products remained where originally located (usually in their own meat-free section outside the meat aisle) without changes to their position. The intervention consisted of moving 26 uniquely barcoded meat-free products within four meat-free categories (50% sausages, 30% burgers, 12% meatballs, and 8% mince) from their usual section into a newly created meat-free bay within the meat aisle. This new bay of meat free products covered the whole bay, including top, middle and bottom shelves. This meat-free section replaced a bay where either meat was previously sold (16 stores), or where the bay previously contained chilled condiments (three stores) or was unused (one store). The meat-free bay was the end bay in the meat aisle in 13 stores and in the middle of the meat aisle in seven stores.  Phase II  The intervention was initially planned by the retail partner for 12 weeks until April 2019 (referred to as intervention Phase I). However, 12 of the 20 intervention stores continued the Phase I intervention until December 2019. For the remaining 8 intervention stores, the retailer intensified the intervention (referred to as intervention Phase II) in the week commencing 28th July 2019 by adding a second bay of meat-free products into the meat-free bay within the meat aisle. This second bay of meat-free products included additional meat-free products (e.g. falafels, sausage rolls, tofu, slices) which were stocked alongside the previous 26 products included in the Phase I intervention. These eight stores were selected by the retailer based on each stores having sufficient meat-free products in stock for the second bay. The Phase II intervention continued in these eight stores until December 2019. |
| 4. The reader would, I'm sure, welcome a bit more description of "Acorn categories" (l.159) to help understand more clearly the metrics used to define store characteristics. | We apologise for the lack of information about the Acorn categorisation system. Regrettably, this source of data used by the retail partner (and many other companies) is private, which means the classification system and methods used are their own intellectual property therefore we have no access to this information. But we have tried to include a few more details of the data sources used by Acorn and a reference to their user guide.  It is also worth noting that these demographic characteristics provided by Acorn (Table 1) do not differ between intervention and control stores, therefore their influence in our results would be minimal. | Methods Page 8:  “The retailer also provided data on demographic characteristics related to the surrounding population. This data was originally derived using the Acorn categorisation system which uses postcode data as well as commercial and governmental data to classify geographical areas across the UK[18].” |
| 5. This reader (at least) would like to see the statistical analysis plan. It is highlighted in the text (l.164-165) but does not seem to have been made available. This is particularly important as the analysis you present in the manuscript deviates in several areas from that in your pre-specified protocol. | We have included the SAP. In response to other reviewers, this new version of the SAP includes the details of the Phase II evaluation which were not initially included |  |
| 6. The fidelity evaluations are clearly important not only to help interpret the study but also to aid wider implementation (i.e. lessons learnt). The protocol suggests that you planned to collect a lot of detailed information about stocking and positioning of meat-free products (including photographs) and the manuscript summarises this as "to assess whether the intervention was implemented as planned". This is critical information that allows the reader to understand more about the implementation of the intervention. The 5 lines on fidelity in the result section provide insufficient and non-systematic detail on this important issue. It's not clear how the reader is supposed to interpret a finding such as "with one store missing 10 meat-free products". Was any of the fidelity information used to inform additional sensitivity analysis? | We apologise for the lack of information and detail regarding the fidelity evaluation. We have added more details in the Methods as well as Results. We didn’t conduct any sensitivity analysis as the results of these fidelity checks suggested that the intervention had been generally implemented as planned. As we discussed, all 20 stores moved a meat-free bay into the meat aisle although some stores missed a small number of products and one store missed 10 products of the 26 products originally designated. We have added a new supplementary table with more details of the fidelity evaluations. | Methods Page 10-11  Results Page 19  Supplementary Table 8 |
| 7. Table 1 is hard to interpret as the categories have not been clearly defined. | See our comment above about the Acorn categories. |  |
| 8. The August 2017-January 2019 trend analysis (supplementary Figure 1) is interesting. I am surprised that while you have noted in the text that the intervention and control stores "followed similar trends" you did not report that there is a clear and consistent difference between the groups over the entire 17 month period i.e. the control stores sold ~1-200 more units of meat than the intervention stores. To me this suggests that the matching didn't work perfectly. | As pointed out by the reviewer, there could be some differences that may appear important when looking at the trends but we aimed to understand these differences in more detail. Firstly, we didn’t find significant differences in demographic characteristics between intervention and control stores (Table 1).  Figure 2 shows the ITS analysis in which sales (units, £) of meat-free items are completely overlapping and show the exact same trend pre-intervention. However sales of meat products in Figure 2 and Suppl Fig 1 showed small absolute differences but a very similar trend pre-intervention. We tested these differences in sales between intervention and control stores over the baseline period (w/c 2 September 2018 to w/c 18 November 2018) and January 2018 and 2019 periods which showed no statistically significant differences (see Supplementary table 2). Therefore we are confident that the selected stores were fairly comparable with regards to demographic characteristics, absolute sales of meat and meat-free products and trends in sales pre-intervention. |  |
| 9. Some of the changes identified in meat-free product sales are tiny - for example in Phase 1, meat-free mince changed from 24 units at baseline to 27 units (while the control dropped from 25 to 24). This shows up as a big effect in Figure 1 but I would suggest you consider more carefully how this is reported. Clearly the size of the change for meat-free burgers and sausages is more substantial. | We agree that the estimate around meat-free mince appears much larger than what it was in absolute terms, but it’s worth noting that the CI around this estimate is also less precise than other categories such as sausages. On the other hand, it’s also worth noting that small change (3 units) can be important given baseline sales are also low (24 units). In any case, our conclusions are mostly based on the overall meat-free or meat categories rather than the individual categories (mince, etc) to avoid relying on changes that can be very small in absolute terms. |  |
| 10. You need to be more consistent (or at least systematic) in the way you report the primary results that I assume come from the pre-specified Phase 1 of the study. For example, why in l.242-244 to you switch from your pre-specified primary outcome "change in weekly sales (units)" to "sales (£ per store per week)"? It is not appropriate to switch outcomes in this manner. | We apologise for this error, as we forgot to specify that those figures corresponded to Suppl Table 4 which showed changes in secondary outcomes.  The primary outcome was not switched and we have revised all results to make sure we have kept this consistent. | Page 12 |
| 11. The primary outcome of the study is clearly pre-specified in the protocol and repeated in the manuscript as "change in weekly sales (units)". In your manuscript you report your primary outcome (units sold) for most of Phase 1 but then use a different outcome "£ per store per week" for Phase 1 time series analyses and all Phase 2 analysis (including the very eye-catching Figures 2 and 3). Is there a reason that you have switched away from your pre-specified primary outcome for these analyses - I could not find a justification in the manuscript and again I really do not think that it is appropriate to switch outcomes in this manner without a very solid justification. It seems to me that you should reproduce Figures 2 and 3 (and the associated text) using your pre-specified primary outcome (units) and these should be the primary results that you report in this paper. | The primary outcome analysis compared changes in units sold per week of total meat products between intervention vs control stores during the Phase I intervention period using a hierarchical negative binomial model.  The ITS model was a secondary outcome analysis and we used £ sales as this metric is more normally distributed than unit sales. However, our statistician has now looked into this issue and was able to plot the ITS models using unit sales to match the primary outcome. These new ITS figures using unit sales completely agree with what we had shown previously so we have left those in the main article and present the ITS figures in £ sales in Supplementary figure 2. | Figures 2 and 3  Supplementary Figure 2 |
| 12. The opening paragraph of your discussion that reports your primary findings is currently hard to interpret without access to analysis reporting your pre-specified primary outcome (units). | As explained above, we have now included our ITS analysis in unit sales to match the primary outcome analysis also in unit sales. However, our conclusions and results do not change and remained consistent. |  |

Reviewer #3: This study aimed to evaluate the effect of positioning meat-free substitutes in the same aisle as the meat products. This study makes an important contribution to the literature. There are aspects of the manuscript that require further clarification and additional details. I have also outlined some methodological/presentation suggestions for the authors to consider.

| Reviewer comments | Authors response | Changes made |
| --- | --- | --- |
| 1. The intervention could be described in greater detail in the abstract, introduction and methods sections in terms of its relevance to existing literature. It is described as increasing the prominence of the positioning of meat-free products but it is unclear how this relates to existing literature which largely describes in-store prominence as being front of store, end-of-aisle and checkouts. The novelty of this study is the revised shelf positioning of the products but this point doesn't appear to be adequately explained in the manuscript, particularly the positioning of the meat-free products along the aisle in relation to the meat products (front, mid, end of aisle and if each meat-free alternative was co-located with the meat versions or whether the meat-free section was altogether). Also whether the positioning of the meat-free products was at eye-level, or bottom shelf or above eye-level. Detailing these positioning factors of the intervention in greater detail could help improve the interpretation of findings (i.e. why some meat-free products sold more than others and why meat product sales did not decline) and aid future intervention design. | We thank the reviewer for their supportive comments and have tried to make all the clarifications in the manuscript.  We have provided some more details of the intervention in the abstract although we are constrained by the word limits. We have tried to include more details and context in the methods so that the novelty of this intervention (repositioning meat-free in a bay next to similar meat items) is clearly stated and the results can be interpreted accordingly. | Methods Page 7  “The intervention was implemented in 20 stores on the week commencing 27th January 2019, for 12 weeks. In the control stores, meat-free products remained where originally located (usually in their own meat-free section outside the meat aisle) without changes to their position. The intervention consisted of moving 26 uniquely barcoded meat-free products within four meat-free categories (50% sausages, 30% burgers, 12% meatballs, and 8% mince) from their usual section into a newly created meat-free bay within the meat aisle. This new bay of meat free products covered the whole bay, including top, middle and bottom shelves. This meat-free section replaced a bay where either meat was previously sold (16 stores), or where the bay previously contained chilled condiments (three stores) or was unused (one store). The meat-free bay was the end bay in the meat aisle in 13 stores and in the middle of the meat aisle in seven stores.” |
| 2. This point links to that above in terms of description of numbers of products available. Greater availability/variety of products has been shown to increase sales of these products in previous literature. It would therefore be helpful for the reader to understand whether there was an increase in the availability/variety of meat-free products as part of the intervention or whether the same number of products were available at baseline, Phase I and Phase II, and how these numbers compared to the control stores. Additionally, what were the numbers/proportion of products available (both meat-free and meat) in the intervention position (both Phases) and in the pre-intervention and the control conditions; were these the same across all intervention stores? (Lines 121-126) | We have clarified this point in the Method section (see above). A total of 26 uniquely barcoded meat-free products were positioned within the meat aisle, without increasing the number/variety of products or introducing new ones.  We have also clarified the Phase II intervention, where the same 26 meat free products were available together with a second bay of other of meat-free products. For both phases, the products were repositioned from other parts of the store, rather than being new introductions to the stores. So we believe that the trial did not result in a change in the ratio of meat to meat-free products available. However, we were unable to access data from the retailer to conduct a specific analysis of product availability as this would have required substantial data extraction resources on their part.  But we understand this is an important consideration and we have added a limitation in the discussion about the lack of detail on the proportion of meat and meat-free products available. | Discussion page 22  “We were not able to access any information regarding availability of products to understand if there were changes in the proportion of meat-free and meat products available on display which could also explain the results.” |
| 3. There is no description of the control condition in the methods section. Providing these details will enable the reader to better understand the intervention design, particularly the in-store and shelf positioning, and number, of meat-free and meat products available for both intervention conditions and the control condition. A full description of the control condition (and any variation across the 88 stores) should be added to the methods section. Also where were intervention and control stores located? | We have included more details of the control conditions, for which the meat-free products remained where originally located.  The intervention and control stores were located across the UK. | Methods page 6 and 7  “In the control stores, meat-free products remained where originally located (usually in their own meat-free section outside the meat aisle) without changes to their position” |
| 4. There are no sample size calculations provided for the experiment. The existing body of literature suffers from a lack of description of power calculations that account for clustering at the store level and this is an essential addition for new studies in this field, particularly for sizeable studies such as this one. It would also be helpful if the authors expanded on the reason for varying numbers of control stores; this is alluded to as a strength of the study in the discussion but no reference or clear rationale is provided. | We apologise for the lack of detail with regards to the power calculations.  We didn’t conduct a power analysis as indicated in our pre-published protocol. Instead, we followed the conduct and evaluation of a natural experiment where the retailer chose to test the intervention in 20 stores (with a sample of matched control stores) with the view of estimating the effect sizes for a future trial. However the retailer did not continue their plans to conduct a larger trial.  We agree that more rigorous designs would be preferable, but as the reviewer is probably aware, the barriers to conducting this type of study in a true field setting are enormous and we feel that this study, closer to a natural experiment than a controlled trial still represents a substantial addition to the academic literature. We have included a sentence to reflect this in the methods section. | Page 8  “Following the conduct and evaluation of a natural experiment, a power analysis was not conducted and the final number of trial stores with matched control stores was chosen by the retailer” |
| Abstract/Introduction:  5. Line 74-78: These sentences could be more specific in terms of my key points 1 and 2 above and how this study relates to existing literature on store/shelf prominence and the marketing practices used by supermarkets. The reference to co-location of complementary products seems fundamental to the intervention design and it would be helpful if this was more clearly explained and referenced by similar studies (e.g. Foster 2014 AJCN and de Wijk 2016 Plos One - probably others from marketing literature). | We thank the reviewer for highlighting this issue and providing relevant literature examples. We have provided a few more detail in Abstract but expanded a bit more on the Introduction with a new reference. | Page 4 and 5  “Systematic reviews which have drawn on broader evidence relating to interventions to shape food purchasing have found moderate to low quality evidence that product placement strategies (e.g. availability or prominent positioning in end-of-aisles, checkouts or at eye-level) implemented in food retail environments can influence dietary behaviours.[13, 14] Also, ‘cross-category merchandising’ (where complementary products are co-located in a store) is an established sales mechanism to increase sales.[15, 16]” |
| Methods:  6. Line 94 - it would be helpful to additional details about the store with two weeks of missing data. Was it intervention/control store? When did the two weeks fall in terms of seasons and the intervention implementation? There is great variability in retail sales weeks so taking the week before and after may not always be the best approach. If it was a control store - would have imputing from an average of the other control stores been an alternative approach? | We have reworded this section and added more details to clarify this. This was one of the intervention stores, which was missing data for 2 non-consecutive weeks at the end of August 2018 and beginning of September 2018 (before the intervention period).  We apologise for the mistake in the text but we actually used the average of the whole month to impute each missing week. We have corrected this. | Page 5  “For one store which had missing data for two non-consecutive weeks in August and September 2018 (0·01% store-weeks), values were imputed using the average of each month.” |
| 7. Lines 104-108; lines 158-161 - The authors conclusions about potential impact on inequalities is based on a measure that of socioeconomic status (SES) that may be unfamiliar to journal readers. Additional details about the affluence measure and how it relates to more typical measures of SES used by researchers would improve understanding and relevance of the associated findings. Additionally, the descriptions of the other variables to determining store matching and confounding variables could be improved to enable clear interpretation of these variables. (i.e. was is meant by meat-free sales performance - total, % sales, alternative? Can examples of store size be provided? What is average affluence? What is the cut-off for young and old? What is meant by average urban, more or less urban?) | We apologise for the lack of information about our SES categories using the Acorn system. Regrettably, this source of data used by the retail partner is private and we have limited information about the methodology used for classifying the store demographics. But we have tried to include a few more details of the data sources used by Acorn and a reference to the user guides which includes details of their customer segmentation.    We have also clarified what is meant by meat-free performance (sales of meat-free products) before intervention and the other variables used to select the stores. | Methods Page 6  “The stores were selected to have a broad spread with regard to the following characteristics: affluence (based on top 2 Acorn consumer classification categories within a 10 minute drive)[18]; sales of meat-free products over a 12 week period ending 25 September 2018; and store size (large (>50,000 sq ft), medium (35-50,000 sq ft), small (<35,000 sq ft).” |
| 8. Lines 109-117 - Use of a computerised matching system provides an objective methods for matching which is beneficial - though reliant on the supermarket rather than the research team. It is difficult to understand how the research matching linked to this matching through the supermarket store details system. Could this description be revisited and a rationale provided. | Unfortunately we didn’t have access to any detail about the matching process which we have tried to describe in as much as possible in the text. We relied on the retailer analytics software to come up with a list of control stores matching the intervention stores based on patterns of meat-free purchases as well as store size and affluence based on Acorn categories. We have shown in Table 1 that the demographic characteristics and trends in sales before the intervention (Suppl Figure 1, Suppl Table 2) are comparable between intervention and control stores. |  |
| 9. Lines 140-146 - would be better placed in another sub-section as they don't directly relate to the intervention description. | We have moved this section to the beginning of statistical analysis as it aims to explain the different phases of the intervention and their baseline periods. |  |
| 10. Related to key-points 1 and 2, it would be helpful if sensitivity analyses were conducted to assess differences in changes to availability of meat-free and meat products across intervention/control stores. (i.e. lines 123-125 state that meat-free replaced meat products suggesting potential decrease in meat product availability and if proportions available altered across stores this additional information about differential intervention effects would make a useful contribution to existing literature). | See our comment above about the availability or changes in number/proportion of meat and meat free products. Unfortunately this information was not provided and we are unable to perform any sensitivity analysis. |  |
| 11. Lines 205-208 - could further details about the measures used to assess fidelity be outlined in this section and a table of the assessments be provided in the results section/supplementary materials? These additional details again provide a helpful addition to previous work. | We have expanded on the description of the fidelity evaluations and included a new table in Supplementary material. | Supplementary table 8 |
| Results:  12. Table 1 - could size of store be added here too? It would be interesting to view the differences in store size across the study stores. | We have included this information in Table 1. | Table 1 |
| 13. Suggest including Supplementary Table 3 in the main body of the results because this contains important detailed information. | Most of the information covered in Suppl Table 3 is presented in Figure 1 so we think there will be a lot of unnecessary overlap but we will leave it to the editor to decide if they want Figure 1 or Suppl Table 3 instead in the main paper |  |
| 14. Figures 2 and 3 seem to be missing a key - difficult to interpret without this when in black and white print. Also, it would be helpful to see the longer-term effect of the intervention in the interrupted time series analyses. Could you test the effect again at time point Nov 2019 not just at the time of implementation? | We have included a key in Figures 2 and 3  Our dataset only runs until Nov 2019 otherwise we would have included all available data in the ITS as we specified in our protocol |  |
| Discussion:  15. Related to key-points 1 and 2, it would be helpful if the discussion reflected the potential differential effects of positioning and availability and how this relates to existing literature. | We have included a limitation point in our discussion about this issue of availability as stated above |  |
| 16. Lines 39-42 (page 20) these analyses are important to have conducted but the interpretation here seems a little inflated because low affluence stores were combined with mid affluence and these were taken at the store rather than household/individual level. I would recommend revising this sentence in light of these factors. | We have reworded this phrase to avoid implying that the associations were at the individual level, and to reflect that the lower affluence group also includes average affluence | Discussion page 21  “Our results provide preliminary evidence that the intervention has potential to be particularly effective in lower and middle affluence populations…” |
| References:  17. References 17 and 18 appear to be the same reference. It would be beneficial for the reader if additional access details were added (i.e. weblink and date accessed if online or full details of author and publisher of only available in print). | We have revised these references and provided web link |  |
