# Supplementary material for: Estimating the effect of moving meat-free products to the meat aisle on sales of meat and meat-free products: A non-randomised controlled intervention study in a large UK supermarket chain
Source: PLoS Med. 2021 Jul 15;18(7):e1003715. doi: 10.1371/journal.pmed.1003715 (PMC8321099; doi:10.1371/journal.pmed.1003715)
Supplement: Attachment — Submitted filename: Reviewer comments.docx [file pmed.1003715.s005.docx]

"Estimating the effect of moving meat-free products to the meat aisle on sales of meat and meat-free products: a non-randomised controlled intervention study in a large UK supermarket chain" (PMEDICINE-D-21-00493R2)

*Editorial comment*

For total clarity and scientific transparency I would like to make one further request. They have (as is good practice) noted with dates the amendments/updates they have made to their protocol (following our earlier review comments). However they have not done this for their statistical analysis plan (SAP). Given the importance of the SAP I think it would be appropriate for them to note (with dates) the changes they have made to this document during the latter stages of the study.

**We have updated the SAP to include dates when changes were implemented**
